# Adipokinetic hormone signaling in the malaria vector *Anopheles gambiae* facilitates *Plasmodium falciparum* sporogony

**DOI:** 10.1038/s42003-023-04518-6

**Published:** 2023-02-13

**Authors:** Vincent O. Nyasembe, Timothy Hamerly, Borja López-Gutiérrez, Alexandra M. Leyte-Vidal, Heather Coatsworth, Rhoel R. Dinglasan

**Affiliations:** Department of Infectious Diseases and Immunology, College of Veterinary Medicine & Emerging Pathogens Institute, University of Florida, 2055 Mowry Road, Gainesville, FL 32611 USA

**Keywords:** Parasite host response, Animal physiology, Infectious diseases

## Abstract

An obligatory step in the complex life cycle of the malaria parasite is sporogony, which occurs during the oocyst stage in adult female *Anopheles* mosquitoes. Sporogony is metabolically demanding, and successful oocyst maturation is dependent on host lipids. In insects, lipid energy reserves are mobilized by adipokinetic hormones (AKHs). We hypothesized that *Plasmodium falciparum* infection activates *Anopheles gambiae* AKH signaling and lipid mobilization. We profiled the expression patterns of AKH pathway genes and *Ag*Akh1 peptide levels in *An. gambiae* during starvation, after blood feeding, and following infection and observed a significant time-dependent up-regulation of AKH pathway genes and peptide levels during infection. Depletion of *AgAkh1* and *AgAkhR* by RNAi reduced salivary gland sporozoite production, while synthetic *Ag*Akh1 peptide supplementation rescued sporozoite numbers. Inoculation of uninfected female mosquitoes with supernatant from *P. falciparum-*infected midguts activated AKH signaling. Clearly, identifying the parasite molecules mediating AKH signaling in *P. falciparum* sporogony is paramount.

## Abstract (web summary)

*Plasmodium falciparum* infection activates adipokinetic hormone signaling and lipid mobilization in the *Anopheles gambiae* mosquito vector to promote sporogony.

## Introduction

Malaria is an important vector-borne disease caused by *Plasmodium* parasites, with more than 400,000 deaths and nearly 200 million cases annually^1^. *Anopheles* mosquitoes transmit *Plasmodium* parasites from human to human via an infectious bite. The obligatory mosquito stages of the malaria parasite represent a critical step in the complex life cycle of this pathogen. When adult female *Anopheles* mosquitoes ingest sexual stage *Plasmodium* gametocytes during blood feeding, the fully mature gametocytes undergo gametogenesis within the mosquito midgut to generate male and female gametes that fuse to form zygotes^2^. The zygotes then mature into motile ookinetes that migrate from the bloodmeal bolus to the periphery, traversing the midgut epithelial cells to the basal compartment where they form oocysts^3^. Each oocyst develops over a period of 12 to 17 days, expanding and undergoing massive mitotic divisions and morphological differentiation (a process called sporogony). These oocysts eventually rupture, releasing thousands of sporozoites that invade the salivary glands and are transmitted to the next vertebrate host via mosquito saliva deposition and parasite inoculation in the skin during a subsequent bloodmeal^3^. Sporogony is metabolically demanding^4^, and the parasite relies on its host vector as a source for nutrients^5^. Studies have shown that mosquitoes with larger teneral reserves are able to support higher parasite loads and survive longer than those grown with lower teneral reserves (from poor larval diet conditions), clearly indicating that mosquito host resources directly influence vector competence^6–8^. The magnitude and viability of the salivary gland sporozoite load is of great interest in malaria epidemiology as it impacts the success of malaria parasite transmission in a community and is a critical factor in the production of whole sporozoite-based malaria vaccines^9,10^.

Several studies have explored mosquito immune responses and nutrient allocation during the early stages of *Plasmodium* infection^11–15^. However, the underlying mechanism driving resource mobilization during sporogonic development remains poorly understood. It has been theorized that the malaria parasite has co-evolved with its *Anopheles* mosquito vectors to ensure maximum transmission potential for the parasite while minimizing vector fitness costs. Various studies have suggested potential parasite-driven manipulation of vector nutritional reserves and immune pathways in a developmental, stage-dependent manner to facilitate sporogony and enhance transmission potential^11,16–18^. Emerging evidence suggests that the malaria parasite taps into lipid reserves in the mosquito fat body for both its energy and structural needs^5,11–13,19^. Atella et al.^13^ described sequestration of the lipid transport protein, lipophorin, by oocyst stages of the avian malaria parasite *Plasmodium gallinaceum* in *Aedes aegypti* midguts ex vivo. Depletion of lipophorin has been shown to negatively impact the number and size of developing oocysts, the number of salivary gland sporozoites, sporozoite mitochondrial activity, and their mammalian infectivity^11,12,18,20^. A study by Costa et al.^11^ also showed that lipids are taken up by developing *P. berghei* oocysts in *An. gambiae*. Nutrient allocation in *Plasmodium-*infected *An. gambiae* has been suggested to be directed towards egg development at early timepoints following bloodmeal ingestion, with the parasite using the remaining non-egg shuttled nutrients in non-competitive vector-parasite interactions^5,11,12^. Together, these studies emphasize the central role of vector host stored lipids in *Plasmodium* sporogony.

Hormonal involvement has also been implicated in these stored lipid parasite-vector interactions. Depletion of 20-hydroxyecdysone (20E), which is an ecdysteroid hormone that controls metamorphosis, was shown to increase the expression of lipophorin, causing a subsequent accumulation of lipids in the midgut tissues and accelerating *P. falciparum* sporogony^12^. Through a non-competitive process, 20E has also been suggested to regulate nutrient allocation between mosquito reproduction and *Plasmodium* parasite development immediately following an infectious bloodmeal^12^. At the molecular level, nutrient allocation between developing mosquito eggs and parasite development is regulated by microRNAs, with miR-276 acting as a molecular switch in nutrient allocation by fine-tuning amino acid catabolism^5^. Insulin-like peptides (ILPs), which are involved in the regulation of hemolymph nutrient homeostasis^21^, have also been linked to a reduction in the *An. stephensi* innate immune response to *P. berghei* or *P. falciparum* infection^14,15^.

To gain more insight into *Anopheles* neuropeptide-mediated mobilization of stored fuels on *P. falciparum* sporogony, we investigated the role that mosquito adipokinetic hormones (AKHs) play in lipid-related resource allocation and lipid metabolism. AKHs are neuropeptide hormones that mobilize stored lipids and glycogen from the insect fat body during extended flight activity, starvation, pupation, and diapause^22^. AKHs belong to a family of short neuropeptides that consist of 7-10 amino acids with a conserved pyro-glutamate at the amino-terminus, two aromatic amino acid residues at position 4 (typically phenylalanine) and position 8 (tryptophan), and an amidated carboxy-terminus^23,24^. In *An. gambiae* two isoforms of AKHs are present, *Ag*Akh1 (pQLFTPAWa) and *Ag*Akh2 (pQVTFSRDWNAa)^23^. They are synthesized as prohormones and stored in the corpora cardiaca until needed^25^. Prior to secretion, the pro-protein is enzymatically cleaved to a single 8–10 amino acid peptide followed by modification at the amino terminus to pyroglutamic acid and amidation of the carboxy terminus^26^. Both peptides signal through a G protein-coupled receptor protein, *Ag*AkhR^23,27^. Although the AKH pathway is known to mobilize stored lipids, it has not been studied in the context of *An. gambiae* – *P. falciparum* interactions. We hypothesized that *P. falciparum* infection influences *An. gambiae* AKH signaling, lipid metabolism, and mobilization of energy reserves to support the parasite’s metabolic and structural needs. Here we show that AKH signaling pathway transcripts and peptides are differentially expressed in a time-dependent manner during *P. falciparum* sporogony. We then employed RNAi to knockdown the *AgAkh1* transcript, and exogenously injected synthetic *Ag*Akh1 peptide to tease out the role of the mosquito AKH pathway in *P. falciparum* sporogony. We further demonstrated the activation of the AKH signaling pathway in uninfected mosquitoes following inoculation of ex vivo supernatant from *P. falciparum* oocyst-infected midguts.

## Results

### Starvation and blood feeding induces time-dependent AKH signaling in *An. gambiae*

**Fig. 1:**
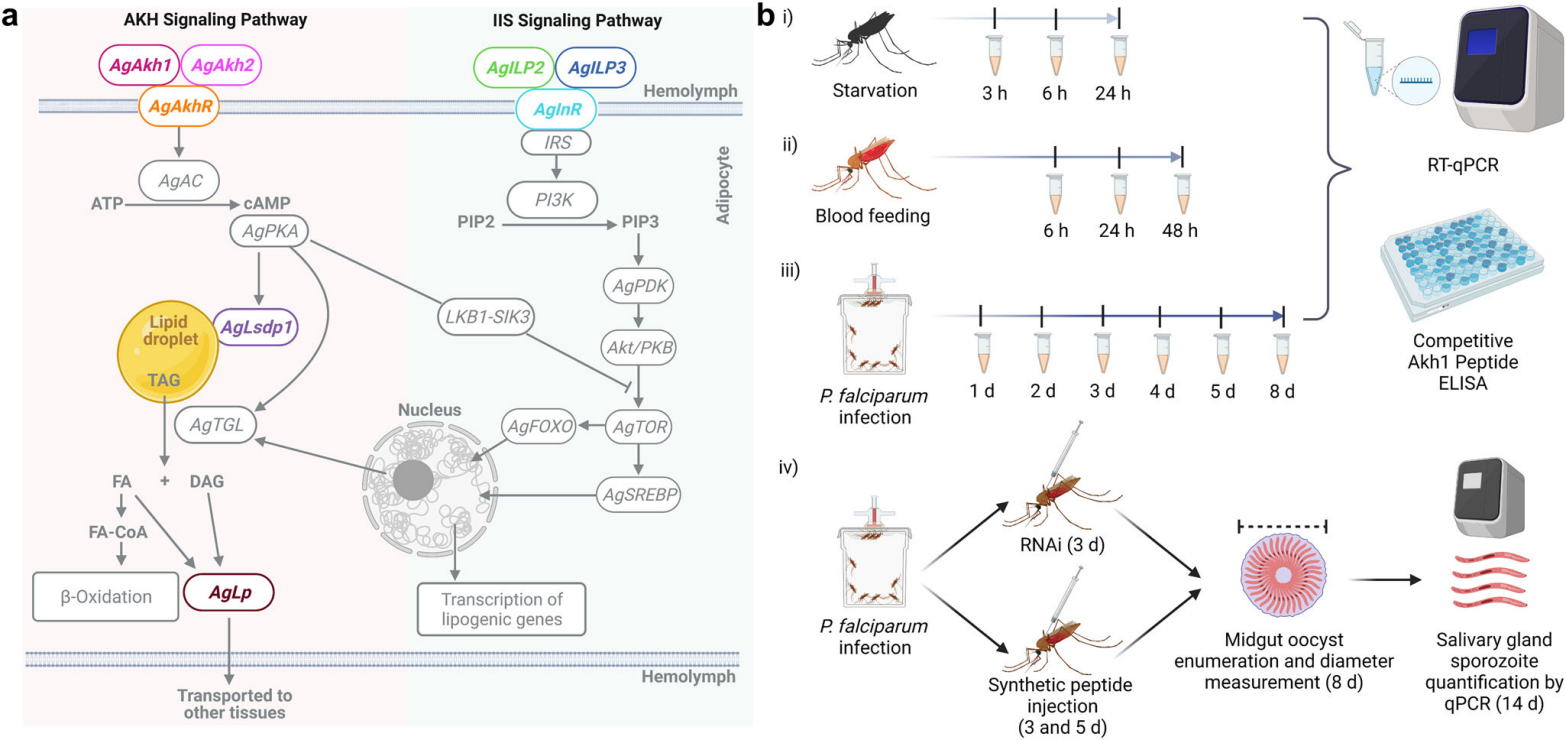
Dissecting the adipokinetic hormone (AKH) signaling pathway in *An. gambiae*. **a** Simplified diagrammatic representation of the proposed AKH signaling and insulin/insulin-like growth factor signaling (IIS) pathway in in *An. gambiae*. Color-coded genes were targeted for transcript quantification using RT-qPCR. *AgAkhR* = *An. gambiae* adipokinetic hormone receptor, *AgLsdp1* = *An. gambiae* Lipid storage droplets surface-binding protein 1, *AgLp* = *An. gambiae* lipophorin, *AgILP* = *An. gambiae* insulin-like peptide, *AgInR* = *An. gambiae* ILP receptor. Other *ILPs* in the IIS pathway that were quantified include *AgILP1, AgILP4, AgILP5, AgILP6, and AgILP7*. **b** Schematic representation of study design depicting starvation (i), blood feeding (ii), *P. falciparum* infection (iii), and AKH perturbation assays (iv). The time points represent the hours of starvation, hours post bloodmeal and days postinfectious bloodmeal when mosquitoes were sampled for analysis. RT-qPCR = reverse transcription quantitative PCR, ELISA enzyme-linked immunosorbent assay. The figures were created with BioRender.com.

**Fig. 2:**
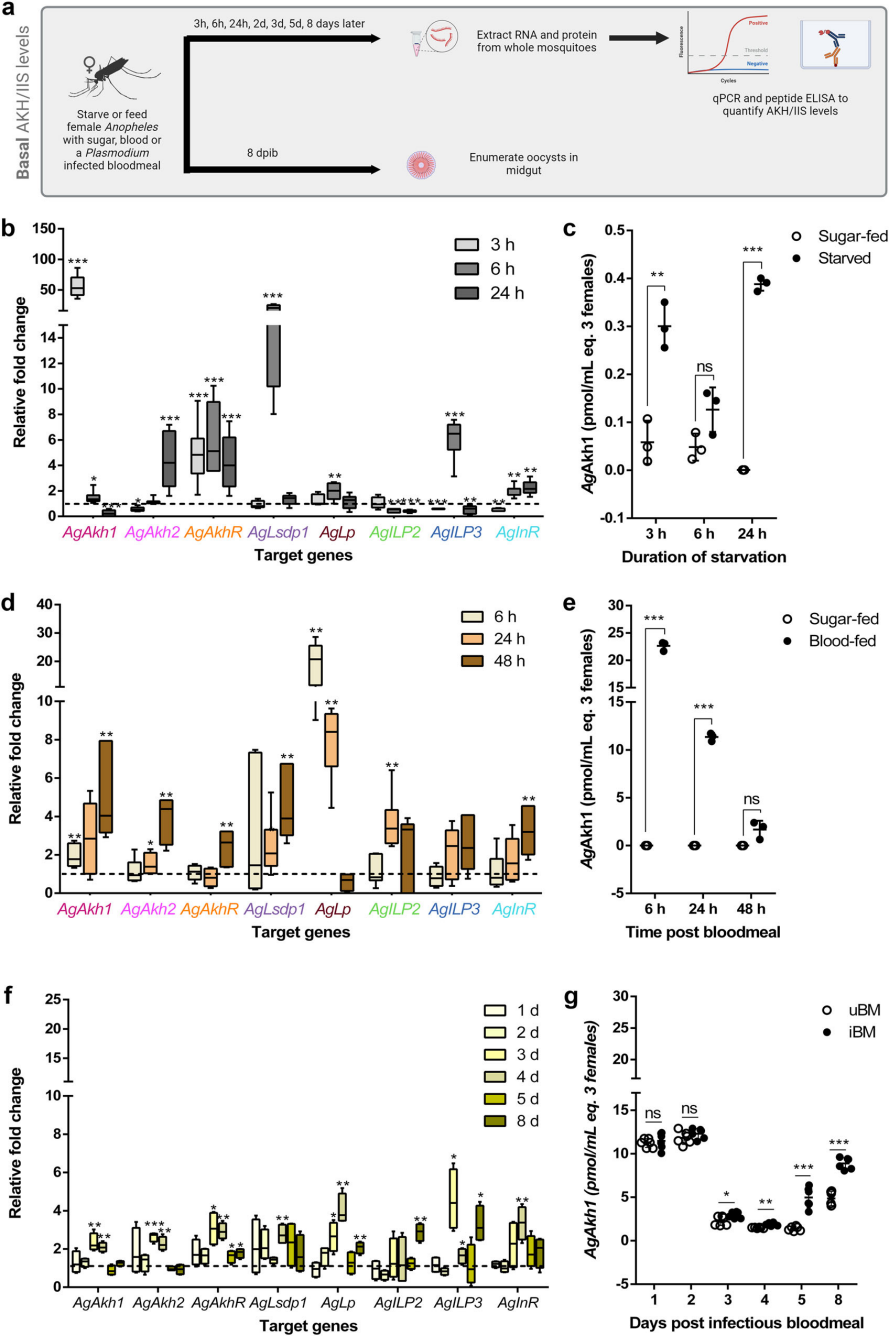
Induction of AKH signaling and insulin/insulin-like signaling (IIS) pathways in *An. gambiae*. **a** Experimental approach for measuring AKH and IIS signaling. **b** Representative replicate showing time-dependent fold change in expression of AKH (*AgAkh1, AgAkh2, AgAkhR, AgLsdp1*, and *AgLp*) and IIS (*AgILP2, AgILP3* and *AgInR*) pathways genes during starvation relative to age-matched sugar-fed females. All expression levels were normalized against the control gene 60 S ribosomal protein L32 (*AgRPL32*). Sugar-fed expression levels were standardized to 1 (dotted line). *N* = 3 pools of 3 mosquitoes for each treatment at each time point. Bars not capped with an asterisk were not statistically different from sugar-fed controls. **c** Representative data showing *Ag*Akh1 peptide titers in sugar-fed and starved female *An. gambiae* at different time points detected by competitive peptide ELISA. *N* = 3 pools of 3 mosquitoes for each treatment at each time point. **d** Representative data showing time-dependent fold change in expression of AKH and IIS pathways genes following blood feeding relative to age-matched sugar-fed females. All expression levels were normalized against the control gene *AgRPL32*. Sugar-fed expression levels were standardized to 1 (dotted line). *N* = 3 pools of 3 mosquitoes for each treatment at each time point. Bars not capped with an asterisk were not statistically different from sugar-fed controls. **e** Representative replicate showing *Ag*Akh1 peptide titers in sugar-fed and blood-fed female *An. gambiae* at different time points post bloodmeal. *N* = 3 pools of 3 mosquitoes for each treatment at each time point. **f** Representative data showing time-dependent fold change in expression of AKH and IIS pathways genes in *P. falciparum*-infected *An. gambiae* relative to uninfected mosquitoes at different time points. All expression levels were normalized against the control gene *AgRPL32*. Uninfected blood-fed expression levels were standardized to 1 (dotted line). *N* = 3 pools of 3 mosquitoes for each treatment at each time point. Bars not capped with an asterisk were not statistically different from uninfected blood-fed controls. **g** Representative data showing *Ag*Akh1 peptide titers in uninfected and *P. falciparum-*infected *An. gambiae* at different time points. *N* = 3 pools of 3 mosquitoes for each treatment at each time point. Error bars represent standard error of the mean. Differences in gene expression between sugar-fed and starved, sugar-fed and blood-fed mosquitoes, and *P. falciparum-*infected and uninfected mosquitoes were detected by independent samples t-test or two-sample Wilcoxon test at 95% confidence interval. * < 0.05, ** < 0.01, *** <0.001, ns = not significant (*P* > 0.05). All statistical analyses were performed at a 95% confidence interval. A total of 9 mosquitoes pooled in groups of three were analyzed for each time point for each feeding status. The assays were repeated three times (Supplementary Fig. S1). A subset of infected mosquitoes *(*N = 20-21) from each replicate were dissected 8 dpi to confirm infection prevalence and intensity using midgut oocyst count as a proxy for infection. *P. falciparum* oocyst prevalence and intensities were 85% and 35 oocysts per midgut, respectively. *AgAkhR* *An. gambiae* adipokinetic hormone receptor, *AgLsdp1* Lipid storage droplets surface-binding protein 1, *AgLp* lipophorin, *AgILP* insulin-like peptide, *AgInR* ILP receptor, uBM non-infectious (uninfected) bloodmeal, iBM infectious bloodmeal, dpib days postinfectious bloodmeal.

Lipid mobilization in insects is known to be mediated by AKHs, which activates triglyceride lipase (TGL) through cAMP-dependent protein kinase A (PKA, Fig. 1a). PKA also phosphorylates lipid-associated proteins such as lipid storage droplet protein 1^28^ (Lsdp1, Fig. 1a). To establish a baseline for the regulation of AKH signaling in malaria vectors, we subjected female *An. gambiae* (Keele strain) to varying lengths of starvation (Fig. 1b (i)) and quantified five transcripts (*AgAkh1, AgAkh2, AgAkhR, AgLsdp1*, and lipophorin (*AgLp*)) in the AKH pathway after each period of starvation (in comparison to their sugar-fed counterparts). The three time points (i.e., 3, 6, and 24 h of starvation) were selected to capture different levels of energy mobilization within a short time frame. *AgLsdp1* was chosen as a proxy for lipid reserve mobilization based on previous studies showing it plays a key role in determining the rate of AKH-mediated lipolysis in several insects, such as *Manduca sexta*^29^, while *AgLp* was chosen to detect lipid trafficking from the fat body^30^. The experimental approach is summarized in Fig. 2a.

As expected, a significant upregulation of *AgAkh1, AgAkh2*, and *AgAkhR* transcripts were detected during starvation (Fig. 2b and Supplementary Fig. S1a); with starved females expressing greater than 50-fold more *AgAkh1* transcripts than their sugar-fed counterparts after 3 h of starvation across the three replicates (W = 81, *P* < 0.001; Fig. 2b and Supplementary Fig. S1A). Variable fold-change was observed in the expression of *AgAkh1, AgAkh2, AgAkhR, AgLsdp1*, and *AgLp* between starved and sugar-fed mosquitoes in the three replicates at different time points (Fig. 2b and Supplementary Fig. S1a). The activation of AKH signaling was further corroborated by an *Ag*Akh1 peptide enzyme-linked immunosorbent assay (peptide ELISA). We observed a significant increase in *Ag*Akh1 peptide levels after 3 h (5.1-fold; t = 4.61, df = 5, *P* = 0.003) and 24 h (516-fold; t = 48.56, df = 5, *P* < 0.001) of starvation (Fig. 2c). Similar increases in peptide levels after 3 and 6 h of starvation were detected in the second and third replicates, although variation in the level of peptide expression was noted across the replicates, owing to the difficulty in controlling for variation in teneral reserves of individual mosquitoes in a pool (Supplementary Fig. S1b). In insects, AKH activity has been shown to be antagonized by the insulin/insulin-like growth factor (IIS) signaling pathway, which regulates the level of trehalose in hemolymph through the induction of lipogenesis^31^. AKH and IIS pathways communicate through liver kinase B1 (LKB1) – Salt-inducible kinase 3 (SIK3) pathways (Fig. 1a). In *An. gambiae*, seven insulin-like peptide (ILP) genes in the IIS pathway have been annotated^32^. However, ILP6 and ILP7 were shown to be duplicate genes of ILP3 (98.7% amino acid sequence identity) and ILP1 (95.5% amino acid sequence identity), respectively^33^, and as such, there are five ILPs including ILP1/7, ILP2, ILP3/6, ILP4, and ILP5^32,33^. We profiled all five *An. gambiae* ILP transcripts and their receptor tyrosine kinase homodimer (*AgInR*; Fig. 1a). We observed a significant decrease in transcript levels of *AgILP2* after 6 and 24 h (2.0-fold, t = 6.59, df = 17, *P* = 0.006; and 2.6-fold, W = 0, *P* < 0.001, respectively), *AgILP3* after 3 h (1.7-fold, W = 0, *P* < 0.001) and 24 h (2.2-fold, W = 4, *P* = 0.014), and *AgInR* after 3 h (1.9-fold, W = 0, *P* = 0.004) of starvation (Fig. 2b and Supplementary Fig. S1a). *AgILP3* transcripts were 5.7-fold higher in starved than sugar-fed mosquitoes after 6 h of starvation (W = 81, *P* < 0.001), while *AgInR* transcripts were 2-fold higher at 6 and 24 h of starvation (*P* = 0.004 and 0.005, respectively; Fig. 2b and Supplementary Fig. S1a). No difference was detected in the transcript levels of *AgILP1, AgILP4, AgILP5, AgILP6 and AgILP7* (Supplementary Table S2).

To establish a baseline for the regulation of the AKH signaling pathway following a bloodmeal, we investigated the expression of the same transcripts as above (Supplementary Table S1) in non-infectious blood fed *An. gambiae* at 6-, 24- and 48 h post bloodmeal (hpbm) feeding (spanning the physiological window of blood feeding from full engorgement with blood until the bloodmeal is almost fully digested), with age-matched sugar-fed females as controls (Fig. 1b (ii)). There was a significant upregulation of *AgAkh1* at 6 (1.9-fold, W = 36, *P* = 0.005), and 48 (5-fold, W = 36, *P* = 0.008) hpbm; *AgAkh2* at 24 (1.5-fold, W = 32 *P* = 0.030), and 48 (3.9-fold, W = 36, *P* = 0.001) hpbm; *AgAkhR* at 48 hpbm (2.4-fold, *P* = 0.009); *AgLsdp1* at 24 (2.4-fold, *P* = 0.019) and 48 hpbm (4.5-fold, W = 36, *P* = 0.005); and *AgLp* at 6 (19-fold, W = 36, *P* = 0.002) and 24 (7.9-fold, W = 36, *P* = 0.005) hpbm (Fig. 2d). Similar upregulation patterns in AKH pathway transcript levels were observed in replicates 2 and 3 (Supplementary Fig. S1c). The activation of AKH signaling was further confirmed by peptide ELISA, which revealed a significant increase in *Ag*Akh1 peptide levels at 6 and 24 hpbm (>1,947-fold change, t = 46, df = 5, *P* < 0.001; t = 47.06, df = 5, *P* < 0.001, respectively) but not at 48 hpbm in blood-fed females, compared to sugar-fed females (Fig. 2e and Supplementary Fig. S1d). In the IIS pathway, only *AgILP2* and *AgInR* transcripts were significantly upregulated at 24 (3.7-fold, W = 36, *P* = 0.006), and 48 (3.2-fold, t = 5.89, df = 11, *P* = 0.005) hpbm, respectively (Fig. 2d and Supplementary Fig. S1c). Akin to what we observed for expression patterns in starved mosquitoes, no significant difference was detected in the transcript levels of *AgILP1, AgILP4, AgILP5, AgILP6* and *AgILP7* (Supplementary Table S2).

### *Plasmodium falciparum* infection induces AKH signaling in *An. gambiae*

To understand how malaria parasite sporogony affects AKH signaling, we infected female *An. gambiae* (Keele strain) with *P. falciparum* (NF54 strain) and sampled at 1-, 2-, 3-, 4-, 5-, and 8-days postinfectious bloodmeal (dpib) to quantify AKH pathway transcripts (Fig. 1b (iii)). The time points were chosen to capture early stages of midgut invasion by the *P. falciparum* ookinetes through mid-to-late stages of oocyst development. Controls, dissected at the same time points, included age-matched females fed a non-infectious bloodmeal. A subset of mosquitoes was dissected from each replicate at 8 dpib to determine oocyst intensity and prevalence as a proxy for infection success. A high oocyst prevalence (86%, 95%, and 90%) and high oocyst intensity (median number of oocysts per midgut = 35, 55.5, and 67) were observed in three replicate experiments. There was a significant increase in the expression of *AgAkh1* (2.3-fold, t = 5.83, df = 7, *P* = 0.009; 2.1-fold, t = 5.12, df = 7, *P* = 0.001, respectively), *AgAkh2* (2.6-fold, t = 6.16, df = 7, *P* < 0.001; 2.3-fold, t = 3.54, df = 7, *P* = 0.004, respectively), and *AgAkhR* (2.1-fold, t = 3.56, df = 7, *P* = 0.019; 2.9-fold, t = 4.36, df = 7, *P* = 0.001, respectively) in *P. falciparum-*infectious bloodfed mosquitoes compared to non-infected females at 3 and 4 dpib (Fig. 2f and Supplementary Fig. S1E). In addition, *AgLsdp1* transcripts were significantly increased at 4 dpi (2.8-fold, t = 2.85, df 7, *P* = 0.004), while *AgLp* transcripts were significantly increased at 3 and 4 dpib (2.6-fold, t = 2.79, df = 7, *P* = 0.019; 4.1-fold, t = 13.29, df = 7, *P* = 0.002, respectively), compared to uninfected females (Fig. 2f and Supplementary Fig. S1e). No difference in *Ag*Akh1 peptide levels were observed between mosquitoes fed on uninfected and *P. falciparum-infected* blood at 1 and 2 dpib across the three replicates (Fig. 2g and Supplementary Fig. S1f). However, *Ag*Akh1 peptide levels were significantly elevated in *P. falciparum* infectious bloodfed *An. gambiae* 3 dpib (1.3-fold; t = 2.574, df = 11, *P* = 0.030; Cohen’s *d* = 5.423), 4 dpib (1.3-fold; t = 5.467, df = 11, *P* = 0.002; Cohen’s *d* = 6.856), 5 dpib (3.6-fold; t = 7.246, df = 11, *P* < 0.001; Cohen’s *d* = 10.175), and 8 dpib (1.8-fold; t = 9.580, df = 11, *P* < 0.001; Cohen’s *d* = 19.174) compared to uninfected *An. gambiae* in replicate 1 (Fig. 2c). A significant increase in *Ag*Akh1 peptide levels was also detected in *P. falciparum-*infected mosquitoes at 3-, 4-, and 5 dpib in replicate 3 and at 8 dpib in both replicates 2 and 3 (Supplementary Fig. S1f). A significant increase in transcript levels in *P. falciparum-*infectious bloodfed mosquitoes were also observed for *AgILP2* at 8 dpib (2.9-fold, t = 8.02, df = 7, *P* = 0.003); *AgILP3* at 3, 4, and 8 dpib (4.6-fold, W = 16, *P* = 0.017; 1.6-fold, t = 2.83, df = 7, *P* = 0.040; 3.2-fold, t = 4.62, df = 7, *P* = 0.016, respectively); and *AgInR* at 4 dpib (3.4-fold, t = 4.81, df = 7, *P* = 0.007), compared to uninfected blood fed mosquitoes (Fig. 2f and Supplementary Fig. S1e). Variable fold change in gene expression of *AgILP1, AgILP4, AgILP5, AgILP6*, and *AgILP7* between infected and uninfected *An. gambiae* at different time points was also observed (Supplementary Table S2).

### Adipokinetic hormone signaling promotes *P. falciparum* sporogony in *An. gambiae*

**Fig. 3:**
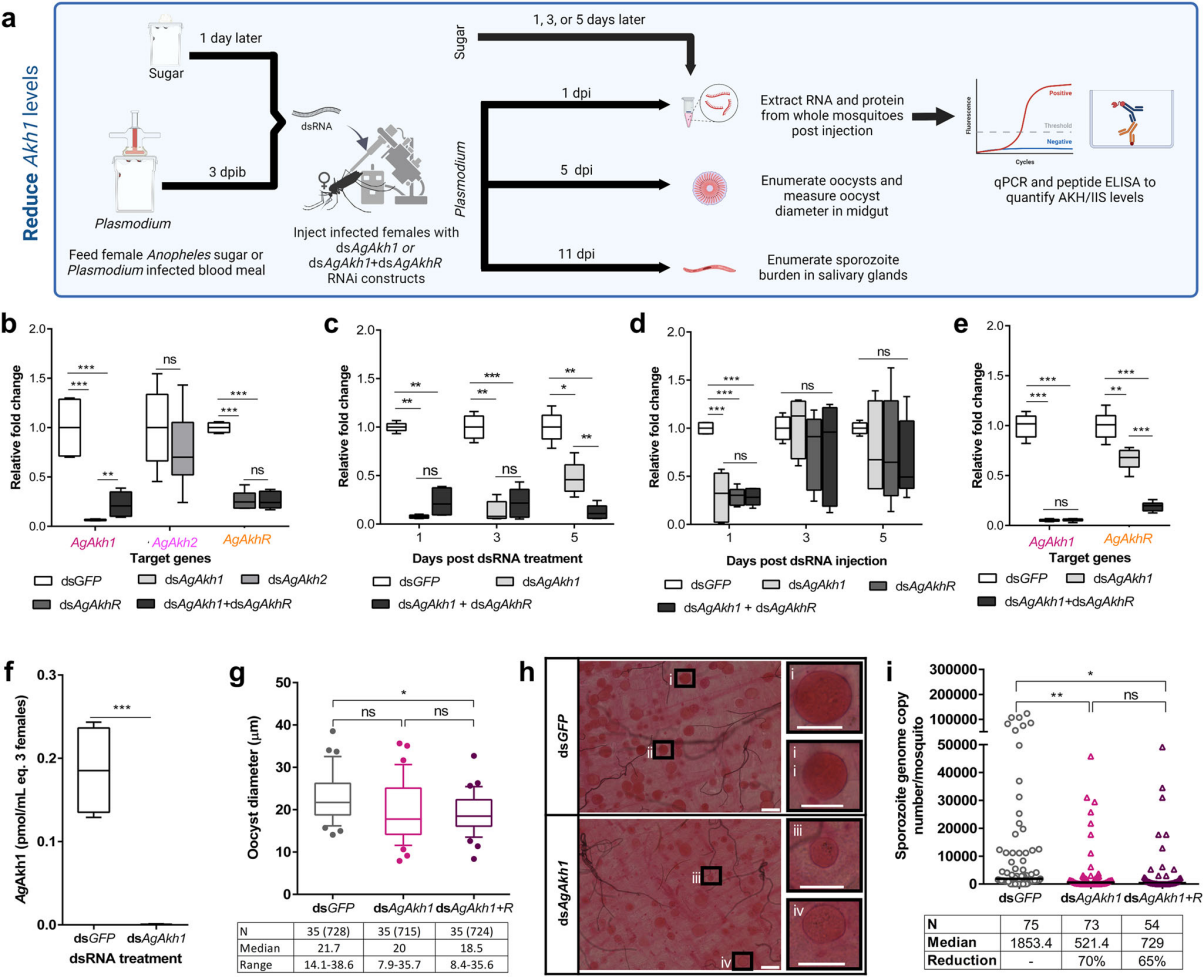
RNAi-silencing of *An. gambiae* AKH signaling reduces *P. falciparum* salivary gland sporozoite infection intensity. **a** Experimental approach to reduce *AgAkh1* levels. **b** Efficiency of double strand RNA (dsRNA) treatment on knockdown of *AgAkh1*, *AgAkh2*, and *AgAkhR* transcripts. *N* = 8 pools of 3 mosquitoes for each treatment. **c** Duration of the knockdown effect of ds*AgAkh1* and ds*AgAkh1* + ds*AgAkhR* treatment on *AgAkh1* transcript levels. *N* = 6 pools of 3 mosquitoes for each treatment at each time point. **d** Duration of the knockdown effect of ds*AgAkh1*, ds*AkhR*, and ds*AgAkh1* + ds*AgAkhR* treatment on *AgAkhR* transcript levels. *N* = 6 pools of 3 mosquitoes for each treatment at each time point. **e** Confirmation of *AgAkh1* and *AgAkhR* transcript knockdown in *P. falciparum*-infected *An. gambiae*. *N* = 9 pools of 3 mosquitoes for each treatment. **f** Reduction of *Ag*Akh1 peptide levels in *P. falciparum*-infected *An. gambiae* following *AgAkh1* transcript knockdown. *N* = 9 pools of 3 mosquitoes for each treatment at each time point. Error bars represent standard error of the mean. **g** *P. falciparum* oocyst diameters in the midguts of *An. gambiae* treated with dsRNA. Numbers shown in brackets are the total number of oocysts whose diameters were measured. **h** Representative brightfield images of stained oocyst-infected midguts of *An. gambiae* treated with dsRNA. Inserts show single oocyst from midguts of ds*GFP* (i and ii) and ds*AgAkh1* (iii and iv) treated female *An. gambiae*. The scale bars in the large images are 50 µm and the scale bars in the inserts are 25 µm. **i** *P. falciparum* sporozoite intensity from salivary glands of dsRNA treated *An. gambiae* detected by qPCR. *dsAgAkh1* + *R* = ds*AgAkh1* + ds*AgAkhR*. dpib = days post infectious bloodmeal, dpi = days post injection. The fold change in gene expression is relative to controls. Figures represent pooled data from three biological replicates. Error bars represent standard error of the mean. Differences in gene expression were detected by independent samples t-test or two-sample Wilcoxon test at 95% confidence interval. * < 0.05, ***< 0.001, ns = not significant (*P* > 0.05).

To establish the role of AKH signaling in *P. falciparum* sporogony, we used RNA interference (RNAi) to reduce AKH transcript levels (Fig. 3a). First, we attempted knockdown of *AgAkh1, AgAkh2* and *AgAkhR* transcripts in uninfected females through intrathoracic injection of corresponding double-stranded RNAs (dsRNA). Whereas knockdown of *AgAkh2* was unsuccessful, we obtained > 90% knockdown of *AgAkh1* (W = 0, *P* < 0.001) and 73% knockdown of *AgAkhR* (W = 0, *P* < 0.001) transcripts compared to ds*GFP* (control)-treated mosquitoes (Fig. 3b). Injection of mosquitoes with a mixture of ds*AgAkh1* and ds*AgAkhR* depleted *AgAkh1* transcripts by 78% (W = 0, *P* < 0.001, Fig. 3b) and *AgAkhR* by 74% (W = 0, *P* < 0.001, Fig. 3b). We further evaluated the duration of ds*AgAkh1*, ds*AgAkhR*, and ds*AgAkh1* + ds*AgAkhR* activity by quantifying transcript levels at 1-, 3-, and 5-days post dsRNA treatment in non-infected mosquitoes. *AgAkh1* transcript levels were significantly reduced during time points that would correspond to early oocyst development at day 1 (92%, W = 0, *P* < 0.001, Fig. 3c) and day 3 (87%, W = 0, *P* < 0.001, Fig. 3c) and mid-oocyst development at day 5 (61%, t = −10.64, df = 7, *P* < 0.001, Fig. 3c) post ds*AgAkh1* treatment, as compared to ds*GFP* controls, suggesting that the duration of knockdown is sufficient to study the impact of *AgAkh1* on *Plasmodium* oocyst development (Fig. 3c). Similar patterns in depletion of *AgAkh1* transcripts at days 1 (77%, W = 0, *P* = 0.002), 3 (78%, t = −9.71, df = 11, *P* < 0.001) and 5 (88%, W = 0, *P* = 0.002) post dsRNA treatment was observed in mosquitoes injected with ds*AgAkh1* + ds*AgAkhR* (Fig. 3c). However, *AgAkhR* transcripts was only significantly depleted on day 1 after treatment with ds*AgAkh1*, ds*AgAkhR* and ds*AgAkh1* + ds*AgAkhR* (t = −7.07, df = 11, *P* < 0.001) (Fig. 3d). No significant difference was detected in the *AgAkhR* transcript on day 3 and 5 (Fig. 3d). Treatment with ds*AgAkh1* and ds*AgAkh1* + ds*AgAkhR* significantly reduced the transcript levels of *AgLsdp1* (t = 3.74, df = 11, *P* = 0.005 and t = 4.16, df = 11, *P* = 0.002, respectively) 1 day post injection compared to dsGFP treated mosquitoes (Table 1). Treatment with ds*AgAkh1* + ds*AgAkhR* also significantly reduced the transcript levels of *AgLsdp1* (t = 4.20, df = 11, *P* = 0.002) 5 days post injection compared to dsGFP treated mosquitoes (Table 1). No significant difference was detected in *AgLp* transcript levels between the dsRNA treatments on day 1, 3 and 5 (Table 1).

**Table 1:** Effect of ds*AgAkh1* and ds*AgAkh1* + ds*AgAkhR* treatment on AKH signaling pathway downstream gene expression.

Days post injection	Target gene^a^	Treatment^b^	N	Mean (Range)	*P* value^c^
**1**	***AgLsdp1***	dsGFP	6	1.00 (0.55–1.45)	
		ds*AgAkh1*	6	0.46 (0.28–0.98)	*P* = 0.005
		ds*AgAkh1* + *AgAkhR*	6	0.40 (0.20–0.59)	*P* = 0.002
	***AgLp***	dsGFP	6	1.00 (0.88–1.12)	
		ds*AgAkh1*	6	0.57 (0.73–0.12)	*P* = 0.176
		ds*AgAkh1* + *AgAkhR*	6	0.76 (0.11–1.53)	*P* = 0.558
**3**	***AgLsdp1***	dsGFP	6	1.00 (0.71–1.29)	
		ds*AgAkh1*	6	0.92 (0.69–1.39)	*P* = 0.832
		ds*AgAkh1* + *AgAkhR*	6	1.12 (0.84–1.30)	*P* = 0.626
	***AgLp***	dsGFP	6	1.00 (0.83–1.17)	
		ds*AgAkh1*	6	0.93 (0.51–1.21)	*P* = 0.896
		ds*AgAkh1* + *AgAkhR*	6	0.88 (0.39–1.21)	*P* = 0.721
**5**	***AgLsdp1***	dsGFP	6	1.00 (0.80–1.20)	
		ds*AgAkh1*	6	0.68 (0.30–1.09)	*P* = 0.093
		ds*AgAkh1* + *AgAkhR*	6	0.42 (0.18–0.56)	*P* = 0.002
	***AgLp***	dsGFP	6	1.00 (0.92–1.08)	
		ds*AgAkh1*	6	1.27 (1.20–1.52)	*P* = 0.176
		ds*AgAkh1* + *AgAkhR*	6	1.23 (0.92–2.00)	*P* = 0.263

^**a**^*AgLsdp1* *Anopheles gambiae* Lipid storage droplets surface-binding protein 1, *AgLp* *Anopheles gambiae* lipophorin.

^**b**^ds*AgAkh* *An. gambiae* Adipokinetic hormone double stranded RNA.

^**b**^Differences in relative gene expression levels between treatments and their respective controls were determined using one-way ANOVA and Tukey’s post hoc test at a 95% confidence interval. *N* = number of replicates.

To disrupt AKH regulation during the sporogonic stages of the malaria parasite, we injected female *An. gambiae* with ds*AgAkh1*, ds*AgAkh1* + ds*AgAkhR* or ds*GFP* three days postinfectious bloodmeal. Since treatment with ds*AgAkhR* alone did not significantly reduce the *AgAkhR* transcript levels beyond day 1 post-treatment, its effect on *P. falciparum* sporogony was not evaluated further. We confirmed knockdown efficiency by quantifying *AgAkh1* and *AgAkhR* transcripts and validating the observed reduction at the peptide level at 24 h post dsRNA injection. We detected a knockdown of *AgAkh1* (95%, W = 0, *P* < 0.001; and 95% W = 0, *P* < 0.001) and *AgAkhR* (35%, t = 6.24, df = 17, *P* = 0.001; and 80%, W = 0, *P* < 0.001) transcripts following treatment with ds*AgAkh1* and ds*AgAkh1* + *dsAgAkhR*, respectively (Fig. 3e), compared to the ds*GFP*-treated group. The *Ag*Akh1 peptide levels were also significantly reduced in ds*AgAkh1* (W = 0, *P* < 0.001; Fig. 3f) *-*treated mosquitoes compared to the ds*GFP*-treated group. The oocyst prevalence was 74%, 85%, and 91% for ds*GFP*, ds*AgAkh1*, and ds*AgAkh1* + ds*AgAkhR* treated mosquitoes 8 dpib, respectively (Table 2). No significant difference was detected in oocyst intensity or prevalence across the three dsRNA treatments (χ^2^_(2, 102)_ = 1.710, *P* = 0.425; χ^2^ = 4.112, df = 2, *P* = 0.128; Table 2). RNAi treatment with ds*AgAkh1* treatment resulted in 1.2-fold reduction in oocyst diameter (Z = −1.863, *P* = 0.082, Cohen’s d = 0.478, Fig. 3h) while ds*AgAkh1* + ds*AgAkhR* treatment reduced oocyst diameter by 1.2-fold (Z = −2.420, *P* = 0.047, Cohen’s d = 0.655, Fig. 3h). Similarly, treatment with ds*AgAkh1* and ds*AgAkh1* + ds*AgAkhR* resulted in significant reduction in salivary gland sporozoite infection intensity. Treatment with ds*AgAkh1* reduced the sporozoite intensity by 70% (Z = − 3.485, *P* = 0.001; Cohen’s d = 0.502, Fig. 3i) at 14 dpib while treatment with ds*AgAkh1* + ds*AgAkhR* reduced the salivary gland sporozoite intensity by 65% (Z = −2.951, *P* = 0.008, Cohen’s d = 0.430, Fig. 3i). The sporozoite prevalence for dsGFP, *dsAgAkh1*, and *dsAgAkh1* + *dsAgAkhR* treatment was 83%, 80%, and 83%, respectively. There was no significant difference in sporozoite infection prevalence between ds*AgAkh1-*, ds*AgAkh1* + ds*AgAkhR-*, and ds*GFP-*treated mosquitoes (χ^2^ = 0.589, df = 2, *p* value = 0.745).

**Table 2:** Modulation of *Ag*Akh1 signaling does not affect *P. falciparum* midgut oocyst intensity.

Study	Treatment^*a*^	*N*	Median oocyst intensity/midgut (range)	Infection prevalence (%)	*P* value^*b*^
**Reduce**	dsGFP (3dpib) - control	46	10 (1–198)	74	–
	ds*AgAkh1* (3dpib)	46	15 (0–110)	85	*P* = 0.194
	ds*AgAkh1* + ds*AgAkhR* (3dpib)	33	21 (0–121)	91	*P* = 0.622
**Increase**	PBS (3dpib) - control	43	56 (0–207)	85	–
	S*Ag*Akh1 (3dpib)	37	24 (0–189)	84	*P* = 0.166
	S*Ag*Akh2 (3dpib)	41	63 (0–192)	95	*P* = 0.467
	PBS (5dpib) - control	55	18 (0–165)	87	–
	S*Ag*Akh1 (5dpib)	59	21 (0–183)	93	*P* = 0.970
	S*Ag*Akh2 (5dpib)	72	14 (0–121)	90	*P* = 0.441

^**a**^ds*AgAkh* *An. gambiae* Adipokinetic hormone double stranded RNA, S*Ag*Akh synthetic *An. gambiae* Adipokinetic hormone, *dpi* days postinfectious bloodmeal.

^**b**^Differences in oocyst intensities between treatments and their respective controls were determined using zero-inflated generalized linear model at a 95% confidence interval. *N* = number of mosquitoes.

### Recombinant adipokinetic peptide hormone increases *P. falciparum* sporozoite infection intensity in *An. gambiae* salivary glands

**Fig. 4:**
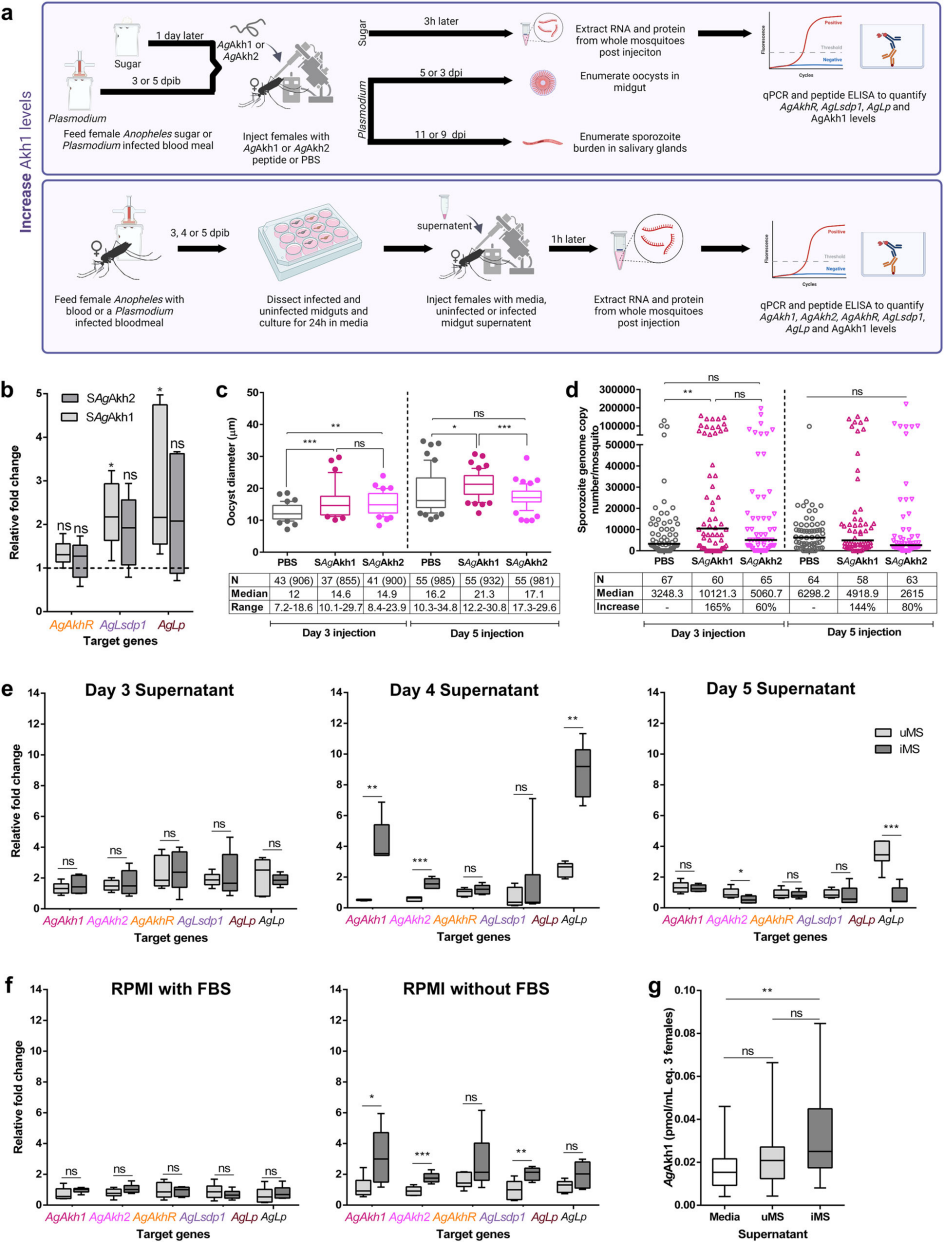
Direct modulation of AgAkh1 peptide levels enhances oocyst maturation and salivary gland sporozoite infection intensity. **a** Experimental approaches to increase Akh1 levels. **b** Effect of synthetic *Ag*Akh1 and *Ag*Akh2 peptide injection on AKH signaling pathway downstream genes. All expression levels were normalized against the control gene 60 S ribosomal protein L32 (*AgRPL32*). Gene expression levels of PBS-injected females were standardized to 1 (dotted line). *N* = 3 pools of 3 mosquitoes for each treatment at each time point. Bars not capped with an asterisk were not statistically different from sugar-fed controls. **c** *P. falciparum* oocyst diameter from the midguts of *An. gambiae* treated with synthetic *Ag*Akh1 (S*Ag*Akh1) and *Ag*Akh2 (S*Ag*Akh2) peptides at 3 and 5 dpi. Numbers shown in brackets are the total number of oocysts whose diameters were measured. **d** *P. falciparum* salivary gland sporozoite intensity from *An. gambiae* treated with synthetic peptides at 3 and 5 dpi detected by qPCR. Differences in oocyst diameters and sporozoite genome copy number were detected using linear mixed-effects models. PBS = phosphate buffered saline, * < 0.05, ** < 0.01, *** < 0.001, ns = not significant (*P* > 0.05). All statistical analyses were performed at a 95% confidence interval. **e** Relative expression of AKH pathway genes following treatment with supernatant collected from *P. falciparum-*infected midguts at 3-, 4- and 5-dpi (days postinjection) following overnight incubation in serum-free RPMI. *N* = 9 pools of 3 mosquitoes for each treatment at each time point. Supernatant from midguts of mosquitoes fed on noninfectious blood meal or RPMI media were used as controls. **f** Relative expression of AKH pathway genes following treatment with supernatant from *P. falciparum-*infected midguts incubated in RPMI or RPMI + FBS at 4-dpi. Supernatant from midguts of mosquitoes fed on noninfectious blood meal or media only were used as controls. *N* = 9 pools of 3 mosquitoes for each treatment at each time point. **g** *Ag*Akh1 peptide titers in female *An. gambiae* treated with supernatant from *P. falciparum-*infected midguts 4-dpi. N = 9 pools of 3 mosquitoes for each treatment at each time point. Gene expressions were normalized to 60 S ribosomal protein L32 (*AgRPL32*). The fold change in gene expression is relative to RPMI or RPMI + FBS media-treated females (standardized to 1). Error bars represent standard error of the mean. Differences in gene expression between mosquitoes treated with supernatant from uninfected and infected midguts were detected by a two-sample Wilcoxon test. Differences in peptide concentration were detected by a Kruskal-Wallis test and a two-sample Wilcoxon test. * < 0.05, ** < 0.01, *** < 0.001, ns = not significant (*P* > 0.05). Figures represent pooled data from three biological replicates. All statistical analyses were performed at a 95% confidence interval. uMS uninfected midgut supernatant, iMS infected midgut supernatant, dpib days postinfectious bloodmeal, dpi days postinjection.

To provide further orthogonal evidence for the role of AKH signaling in *P. falciparum* sporogony, we injected synthetic *Ag*Akh1 (S*Ag*Akh1) and *Ag*Akh2 (S*Ag*Akh2) peptides into mosquitoes (Fig. 4a). First, we tested the potential of these synthetic peptides to mimic natural peptides by micro-injecting sugar-fed female *An. gambiae* with S*Ag*Akh1 and S*Ag*Akh2 peptides and evaluating their effect on the downstream genes *AgAkhR*, *AgLsdp1*, and *AgLp*. Inoculation of S*Ag*Akh1 did not elicit a significant change in *AgAkhR* transcripts (t = 1.82, df = 11, *P* = 0.191, Fig. 4b), with only S*Ag*Akh1 inducing a significant upregulation of *AgLsdp1* (2.2-fold, W = 0, *P* = 0.049, Fig. 4b) and *AgLp* (2.8-fold, W = 0, *P* = 0.017, Fig. 4b) three hours post-injection compared to sterile PBS-injected controls; like the patterns observed in starved versus sugar-fed females. Next, we micro-injected *P. falciparum-*infectious blood-fed female mosquitoes with S*Ag*Akh1 and S*Ag*Akh2 peptides at 3 dpib and 5 dpib. Oocyst diameter and salivary gland sporozoite infection intensity was used to gauge the impact of the synthetic peptides on sporogonic development, while oocyst prevalence and intensity were used as a proxy for successful infection.

Like the RNAi studies, no significant difference was detected in 8 dpib oocyst intensity or prevalence when mosquitoes were injected with both peptides at 3 dpib (χ^2^_(2, 106)_ = 1.906 *P* = 0.386; χ^2^ = 2.814, df = 2, *P* = 0.245, respectively, Table 2) and 5 dpib (χ^2^_(2, 102)_ = 0.793, *P* = 0.0.673;; χ^2^ = 0.423, df = 2, *P* = 0.809, respectively, Table 2). Mosquitoes injected with S*Ag*Akh1 at 3 and 5 dpib had 1.2-fold (Z = −4.045, *P* < 0.001; Cohen’s *d* = 1.340) and 1.1-fold (Z = −2.598; *P* = 0.024; Cohen’s *d* = 0.342) larger oocyst diameters compared to the sterile PBS (control)-treated group, respectively (Fig. 4c). Treatment with S*Ag*Akh1 peptide at 3 dpib resulted in a 165% increase in salivary gland sporozoite numbers (Z = −3.579, *P* = 0.001; Cohen’s *d* = 0.969; Fig. 4d). Treatment with S*Ag*Akh2 at 3 dpib led to a 1.2-fold (Z = 1.254, *P* = 0.004; Cohen’s *d* = 1.192) increase in oocyst diameter (Fig. 4c) but did not result in a significant increase in salivary gland sporozoite numbers (Fig. 4d). The 14 dpib sporozoite prevalence for PBS-, *SAg*Akh1-, and S*Ag*Akh2*-*treated mosquitoes was 79%, 83% and 78%, respectively, 3 dpib, and 88%, 91% and 87%, 5 dpib, and these results were not significantly different (X-squared = 0.39745, df = 2, *p* value = 0.8198, 3dpib, X-squared = 0.6273, df = 2, *p* value = 0.7308, 5dpib).

### *Plasmodium falciparum* oocyst supernatant stimulates AKH signaling

The blood stages of *Plasmodium* parasites have been estimated to export more than 400 proteins (known as the secretome or exportome) into the host erythrocytes or plasma that can modulate host cell physiology and immune response^34–37^. We posited that the rapidly developing oocyst stages of the parasite secrete proteins into the mosquito hemolymph that modulate lipid reserve mobilization. To test this, we dissected midguts from mosquitoes fed on *P. falciparum*-infectious blood at 3-, 4-, and 5 dpib and incubated them overnight in incomplete medium, which corresponds to the different oocyst developmental stages and our previously observed differences in transcript profiles. The midgut supernatants were collected after a 24 h incubation, concentrated, and injected into uninfected 5- to 6-day-old females. Day 8 median oocyst intensity and prevalence were 8.5 oocysts/midgut and 90%, 39 oocysts/midgut and 90%, and 60 oocysts/midgut and 90% for replicates 1, 2, and 3, respectively. Midgut supernatant from infectious blood-fed mosquitoes from 3 dpib did not elicit any change in the expression levels of *AgAkh1, AgAkh2, AgAkhR, AgLsdp1*, and *AgLp* transcripts, while the expression levels of *AgAkh2* and *AgLp* were downregulated 1.7-fold (t = 2.49, df 11, *P* = 0.035) and 4.6-fold (t = −6.34, df = 11, *P* < 0.001), respectively, in mosquitoes injected with day 5 dpib midgut supernatant from infectious blood-fed mosquitoes (Fig. 4e). On the other hand, 4 dpib midgut supernatant from infectious blood-fed mosquitoes elicited significant upregulation of *AgAkh1* (8.5-fold, W = 36, *P* = 0.001)*, AgAkh2* (2.7-fold, t = 6.35, df = 11, *P* < 0.001), and *AgLp* (3.6-fold, W = 36, *P* = 0.002) transcripts (Fig. 4e). The observed activation of AKH pathway genes by 4 dpib with midgut supernatant from infectious blood-fed mosquitoes suggested that signaling to mobilize lipid reserves occurred due to an increased metabolic demand imposed by the rapidly growing parasites.

To test this, we repeated the previous study but only incubated 4 dpib *P. falciparum-*infected midguts in two sets of media, RPMI only and RPMI plus heat-inactivated fetal bovine serum (FBS). Midguts of age-matched females fed on a non-infectious bloodmeal and collected at the same time intervals, as well as RPMI + FBS with no midguts were used as controls. Day 8 median oocyst intensity and prevalence were 60.5 oocysts/midgut and 100%, 44.5 oocysts/midgut and 80%, and 139 oocysts/midgut and 90% for replicates 1, 2 and 3, respectively. As expected, there was no significant difference in the expression levels of *AgAkh1, AgAkh2, AgAkhR, AgLsdp1, and AgLp* when lipid-rich FBS was added, as compared to non-infectious blood-fed controls (Fig. 4f). Injection of female mosquitoes with supernatant from 4 dpib midguts infectious blood-fed mosquitoes without FBS resulted in a significant upregulation of *AgAkh1* (3.2-fold, t = 2.64, df = 11, *P* = 0.029), *AgAkh2* (1.7-fold, 4.89, df = 11, *P* = 0.001), and *AgLsdp1* (2-fold, t = 3.48, df = 11, *P* < 0.001) compared to those inoculated with supernatant from uninfected midguts (Fig. 4f). A significant difference in *Ag*Akh1 peptide concentration was detected between mosquitoes treated with RPMI media and 4 dpib midgut supernatant from infectious blood-fed mosquitoes (W = 147.5, *P* = 0.004; Cohen’s *d* = 0.515), but not between those treated with uninfected and infected midgut supernatants (W = 228.5, *P* = 0.068, Cohen’s *d* = 0.202) (Fig. 4g).

## Discussion

**Fig. 5:**
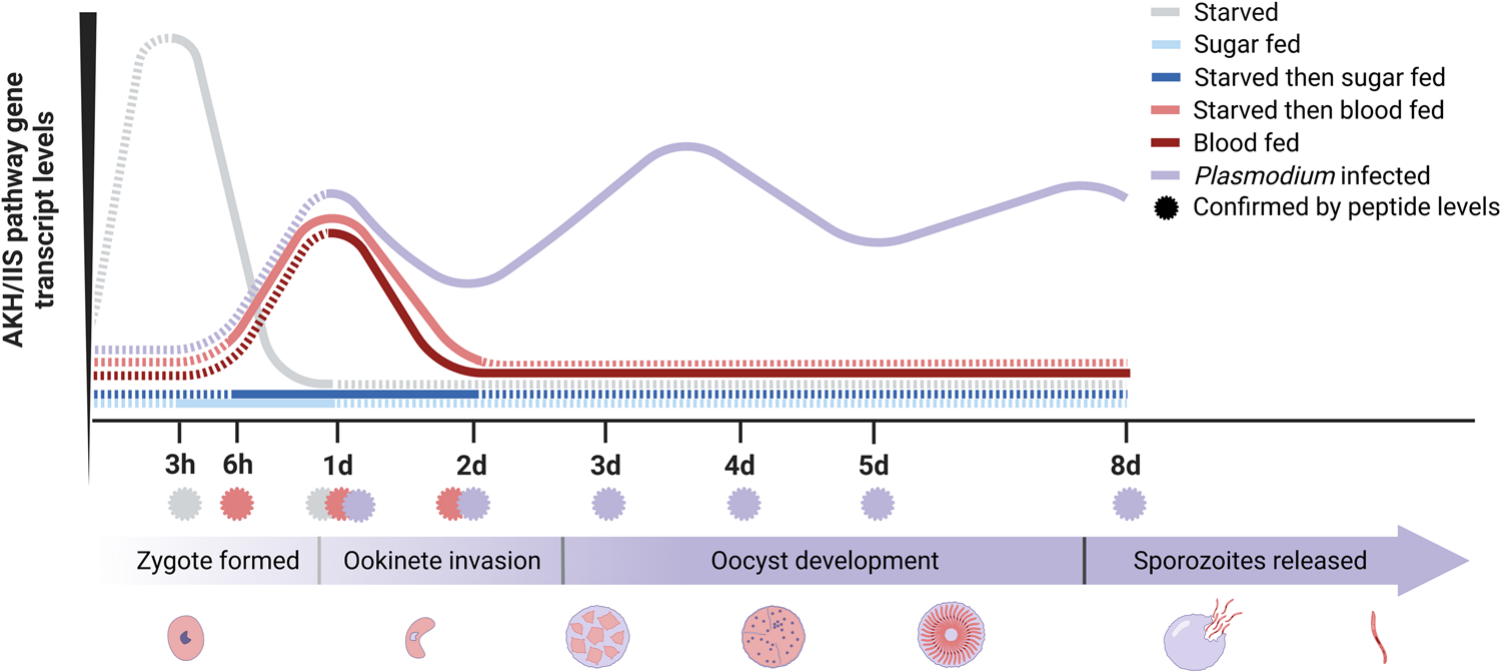
Graphical representation of measured AKH/IIS pathway gene transcript levels across time and experimental conditions. AKH/IIS pathway gene transcript levels increase in starved (light gray) conditions between 3 and 6 hours, as verified by AgAkh1 peptide titers (light gray asterisks), in comparison to the sugar-fed condition (light blue). Between 6 and 48 hours, the starved then blood-fed treatment (light red) increases in AKH/IIS pathway gene transcript levels, as verified by peptide titers at these timepoints (light red asterisks), in comparison to the starved then sugar-fed group (dark blue). AKH/IIS pathway gene transcript levels increase from 3–8 days in the *Plasmodium-infected* blood-fed condition (purple), with spikes coinciding with oocyst development and sporozoite release (pictorially represented under the purple *Plasmodium* development arrow) and verified by peptide titers (purple asterisks), in comparison to the blood-fed only samples (dark red). Proposed but not measured trends for each condition are displayed as color-matched dashed lines.

The role of AKH signaling is poorly understood in the malaria vector *An. gambiae*. Using starvation and blood-feeding assays, we explored the role of the AKH signaling pathway in this mosquito species and its involvement in lipid metabolism and utilization of energy reserves (Fig. 5). During starvation, AKHs mobilize glycogen and lipids from the fat body of several insect species to sustain metabolic processes vital for survival^38–40^. AKHs have been shown to induce lipolysis in the fat body of *Manduca sexta* and *Drosophila melanogaster* through PKA-mediated phosphorylation of *Lsdp1*, thus allowing triglycerides (TAG) to access the hydrophobic lipid droplet surface and hydrolyze TAG to diglycerides and free fatty acids^28,29,41^. Our study shows an upregulation of AKH pathway genes including *AgAkh1, AgAkh2, AgAkhR, and AgLsdp1* in starved female mosquitoes within a 48 h period. *AgLp*, which is involved in trafficking of mobilized lipids, was also upregulated. In addition, *Ag*Akh1 peptide levels were significantly elevated, pointing to the possible involvement of this pathway in energy reserve mobilization. On the other hand, blood-feeding induces a massive metabolic shift geared towards egg production that is accompanied by mobilization and deposition of lipids in developing oocytes^30^. Our study shows a time-dependent increase in the transcript levels of the AKH pathway genes over 48 h post blood feeding; data that are congruent with previous findings by Kaufmann and Brown^23^ describing the expression of *AgAkh1, AgAkh2, and AgAkhR* transcripts in different tissues of blood-fed female *An. gambiae*. The robust expression of *Ag*Akh1 peptide within 24 h post blood feeding indicates a relatively rapid and sustained process, putatively involving enzymatic cleavage of the prohormone triggered by a bloodmeal signal. Our results also revealed time-based downregulation of *AgILP2*, *AgILP3*, and *AgInR* during starvation and upregulation following a bloodmeal, suggesting active involvement of the IIS pathway in hemolymph nutrient homeostasis in the malaria vectors^31^.

*Plasmodium* parasites require nutrients for metabolic processes that support both parasite schizogony (human) and sporogony (mosquito). Whereas nutrient utilization has been extensively studied in the blood stages of the parasite^42^, nutrient utilization during sporogony is poorly understood. 20E and microRNAs have been shown to regulate nutrient allocation between developing eggs and early stages of malaria parasite development in the mosquito following an infectious bloodmeal^5,12^. Here, we demonstrate the activation of mosquito AKH signaling during *P. falciparum* sporogony between day 3 and 8 postinfectious bloodmeal (Fig. 5). These findings underpin the significance of these time points in the biology of *Plasmodium* sporogony. The parasite completes its transition into oocysts within 36 hours of ingestion by *Anopheles*, and initiates the process of sporoblast formation, extensive DNA replication, cell division, and maturation resulting in the production of sporozoites^3^. Unlike the asexual blood stages that rely on glycolysis, the mosquito stages of *Plasmodium* are dependent on the tricarboxylic acid (TCA) cycle, with one input being acetyl-CoA derived from catabolism of free fatty acids^43^. The increased AKH pathway signaling between day 3 and 4 postinfection is, in part, suggestive of the response in the mosquito vector to increased demand of lipids for energy and structural components such as cell membranes by the oocyst. We observed an initial spike in *Ag*Akh1 peptide levels in females fed a non-infectious and infectious bloodmeal within the first 24 hours, in support of the logical notion that mobilization of stored lipids at this time is to support egg development. *Ag*Akh1 peptide levels begin to rise again after 3 dpib, becoming significantly different from the uninfected group at 3-, 4-, 5-, and 8 dpib, suggesting that the developing parasite can influence AKH signaling during sporogony, long after blood feeding and blood digestion. These data suggest that even if the mosquito vector is not able to take another bloodmeal, the maturing oocyst has evolved a mechanism to trigger lipid mobilization.

Studies by Werling et al.^12^ demonstrated increased accumulation of glycerolipids and phospholipids in the midguts of nuclear receptor EcR/USP depleted *An. gambiae*, which is associated with accelerated *P. falciparum* development. Although this was attributed to possible downregulation of *An. gambiae* hepatocyte nuclear factor 4 that controls lipolytic genes^12^, the involvement of other pathways was not ruled out. We demonstrate the involvement of *An. gambiae* AKH signaling in the regulation of *P. falciparum* sporogony. Specifically, depletion of *AgAkh1* and *AgAkhR* transcripts through RNAi resulted in a decrease in oocyst diameter and lower salivary gland sporozoite infection intensity, suggesting an arrest in the development of oocysts. On the other hand, supplementing infected female *An. gambiae* with synthetic *Ag*Akh1 peptide resulted in larger oocyst diameters and higher sporozoite numbers in the salivary glands. Previous studies have shown that giving mosquitoes a second bloodmeal a few days after the first infectious bloodmeal accelerates the rate of parasite development and total salivary gland sporozoite load, possibly due to additional nutrients acquired from the second bloodmeal^44,45^. Therefore, our findings indicate activation of AKH signaling pathway has the potential to increase sporozoite production even in the absence of multiple bloodmeals. Although we were unable to deplete *AgAkh2* transcripts, the addition of synthetic *Ag*Akh2 peptide did not elicit any change in parasite development or sporozoite infection intensity, suggesting its presumed non-involvement. Together, these data highlight a vector host-pathogen dynamic in the use of mosquito lipid energy reserves during sporogonic development, likely a result of a long evolutionary relationship between the malaria parasite and its primary vector (Fig. 5). While our study demonstrates that AKH signaling plays a significant role in *P. falciparum* sporogony, additional studies evaluating the effect of RNAi and synthetic peptide injections at additional time points in the parasite sporogony is necessary to tease out the kinetics of its involvement.

The blood stages of the malaria parasites have been shown to secrete a plethora of proteins into the host erythrocytes and plasma during development^27,28^. These proteins serve as virulence factors that can modulate or evade immune responses, as well as mobilize key resources to support parasite development. For example, *P. falciparum-*secreted rhoptry protein RhopH2 was shown to increase erythrocyte membrane permeability thus allowing uptake of nutrients^46^. In addition, *P. falciparum* Niemann type C like 1 (NPC1L1) protein has been shown to import sterols and maintain *Plasmodium* plasma membrane lipid homeostasis^47^. During sporogony, we show the activation of AKH pathway genes in uninfected mosquitoes following injection of supernatant from infected midguts, similar to patterns of activation of AKH pathway genes that were observed in infected mosquitoes. The fact that the AKH pathway genes were not activated when mosquitoes were injected with supernatant from midguts incubated in FBS-containing media further alludes to the central role played by lipids in parasite sporogony. This could have been caused by the fact that FBS has ample lipid content, hence the parasite did not need to stimulate lipid mobilization. In addition, the activation of the AKH pathway by supernatant from 4 dpib midguts is intriguing. It suggests that the window within which the parasite passively or actively triggers resource mobilization in mosquito vectors is small. This can be attributed to the fact that around this time, sporoblast formation is complete and the sporozoites are rapidly differentiating and forming circumsporozoite protein^3^.

Our data also suggest that lipid mobilization may be a result of heretofore uncharacterized interactions between mosquito tissues and parasite-secreted molecules, or increased lipid mobilization by mosquito in response to parasite infection and represents a significant step towards understanding the potential manipulation of mosquito physiology by the parasite. In the future, it will be important to determine if this interaction is active, i.e., the parasite secretes specific molecules to mobilize vector host lipid stores, or if the molecules (some of which may not be normally secreted) are passively exported or expelled as part of a remodeling process as one oocyst becomes thousands of sporozoites. In the latter scenario, the molecules are then “sensed” by the mosquito, signaling through the AKH pathway. Further studies will be needed to elucidate the identity and role of these molecules in parasite transmission biology. Furthermore, the current study cannot rule out the potential role of mosquito-secreted molecules in response to oocyst maturation.

Our study presents insights into *P. falciparum* sporogony and opportunities to increase sporozoite yield for whole sporozoite-based vaccine development and controlled human challenge studies, which are impacted by the challenge of routinely and cost-effectively producing large numbers of sporozoites at the requisite scale^48^. However, we anticipate that a downside of manipulating this system through the AKH pathway, is that the delicate balance of the natural system is perturbed, which may result in reductions in mosquito fitness and viability, i.e., requiring large numbers of mosquitoes or reducing adult mosquito output in the insectary to ensure large, robust females are produced. This study also lays the foundation for the development of new malaria control interventions, i.e., drugs that can clear a mosquito of oocyst infections or arrest the development of sporozoites within intact oocysts by modulating nutrient mobilization via AKH signaling^49^.

## Methods

### Mosquito colony maintenance

*An. gambiae* (Keele) was used in all experiments. Adults were reared at a temperature of 27 °C with a relative humidity of 75–80% and a 12-h light/dark photoperiod. Larvae were maintained on Koi fish food pellets and adults were offered 10% sugar solution *ad libitum*.

### Starvation and blood feeding assays

Two separate feeding assays were carried out with *An. gambiae* mosquitoes. In the first assay, 5- to 6-day-old adult female mosquitoes with no prior bloodmeal were starved of sucrose solution for 3, 6, or 24 h. Controls included age-matched females drawn from the same mosquito cohort but given 10% sucrose solution *ad libitum*. At each time point, (3-, 6-, and 24 h) nine mosquitoes were sampled. Mosquitoes were either i) pooled into groups of three and deposited in 1.5 mL microcentrifuge tubes containing TRIzol reagent, and immediately stored at −80 °C (for RT-qPCR assays) or ii) pooled into groups of three, snap-frozen in liquid nitrogen and stored at −80 °C (for enzyme-linked immunosorbent assays (ELISAs)). In the second assay, 5- to 6-day-old adult female *An. gambiae* were starved for 6 h, then given either 10% sucrose solution (control) or a human bloodmeal (50% hematocrit in heat-inactivated human serum) offered through membrane feeding assays for 30 min. Similarly, nine females were sampled from each feeding condition at 6-, 24-, and 48 h post bloodmeal and pooled into groups of three. Again, mosquitoes were either stored in TRIzol reagent or snap-frozen in liquid nitrogen. For each feeding assay, we performed three independent biological replicate experiments.

### Plasmodium falciparum infection assays

A culture of *P. falciparum* (NF54) gametocytes was produced as follows^49^. Gametocyte cultures were seeded at 0.5% asexual parasitemia and 4% human hematocrit and were grown under hypoxic conditions in RPMI culture media supplemented with HEPES, hypoxanthine, glutamine, and 10% heat-inactivated human serum. The RPMI media was changed daily during culturing and gametocytes were harvested at day 17. Infected erythrocytes were brought up in heat-inactivated human serum (Interstate Blood Bank, Memphis, TN) plus human RBCs (blood type O^+^) at 1% gametocytemia and 50% hematocrit. Infective blood was delivered directly into glass, water-jacketed membrane feeders warmed to 37 °C. Mosquitoes (6–8 day old) were allowed to feed for 20 min. Fully blood-engorged female mosquitoes were separated and maintained for 14 days on 10% sucrose solution. A sub-set of whole mosquitoes were collected at 1-, 2-, 3-, 4-, 5-, and 8-days postinfectious bloodmeal (dpib) and either preserved in TRIzol for RT-qPCR or snap frozen in liquid nitrogen for ELISA. A separate subset of mosquitoes was collected at 8 dpi for midgut oocyst enumeration. Controls included age-matched females fed on a non-infectious bloodmeal collected under the same sampling regime. At each sampling point, nine females were sampled and processed in pools of three. A total of three biological replicates were obtained for each time point analyzed. The dissected midguts were stained with 0.1% mercurochrome to visualize and quantify oocyst burden. For sporozoite quantification, salivary glands were dissected in 1X phosphate buffered saline (PBS) and transferred individually into 1.5 mL tubes containing 100 µL DNAzol.

### Quantification of vector host gene expression profiles by quantitative PCR

Total RNA was extracted from the starved, sugar-fed, blood-fed, and *P. falciparum-*infected mosquito pools in TRIzol reagent (Invitrogen) according to the manufacturer’s instructions, including a DNase I (Turbo DNase kit^TM^, ThermoFisher) step. cDNA was synthesized from 1 µg of RNA using Superscript IV Reverse Transcriptase and Oligo(dTs) (ThermoFisher).

The expression of AKH pathway genes (*AgAkh1, AgAkh2, AgAkhR, AgLsdp1*, and *AgLp*) and insulin/insulin-like growth factor genes (*AgILP1, AgILP2, AgILP3, AgILP4, AgILP5, AgILP6, AgILP7*, and *AgInR*) were quantified from the synthesized cDNA via qPCR, using *An. gambiae* ribosomal protein transcripts *AgRPL32* as internal controls. Primer pairs were designed using Integrated DNA Technology freeware (https://www.idtdna.com/scitools/Applications/RealTimePCR/) so that at least one oligonucleotide spanned an exon – exon boundary to prevent genomic DNA amplification. Amplicons were designed to be no longer than 150 base pairs, to promote amplification efficiency (Supplementary Table S1). Transcript sequence and exon boundary information were retrieved from VectorBase^50^. Primer specificity was first verified in silico by BLAST comparisons of the oligonucleotide sequences against all sequences hosted by National Center for Biotechnology Information (https://www.ncbi.nlm.nih.gov/). Optimal amplifying and annealing conditions were then tested by gradient PCR for each set of primers. In addition, the primers were tested for amplification efficiency using qPCR. Each qPCR reaction contained 0.75 µL cDNA template, and a reaction master mix containing 6.25 µL SYBR Green Master Mix (Thermo Fisher Scientific) and 0.3 µM of each primer in a total reaction volume of 12.5 µL. The thermal profile used for qPCR was: 1 min at 95 °C; 40 cycles of 15 s at 95 °C and 1 min 60 °C. All qPCR assays were run as technical duplicates and each plate included a no template control. All assays were run on an Eco™ Real-Time PCR System (Illumina).

### Quantification of sporozoite prevalence and abundance by quantitative PCR

To quantify sporozoite burden, DNA was first extracted from parasite-infected salivary glands macerated using sterile plastic pestles. Each tube was then spun down at 12,000 x g for 10 min to remove tissue debris and DNA was extracted following DNAzol’s manufactures’ instructions, with the addition of 6 µg of glycogen before ethanol precipitation (used as a DNA carrier).

For sporozoite quantification, the primers and hydrolysis probe targeting the *P. falciparum* single copy gene glutathione reductase (*PfGR*, PF3D7_1419800)^51^ were designed using Integrated DNA Technologies’ Real Time PCR tool (https://www.idtdna.com/scitools/Applications/RealTimePCR). Forward and reverse primer sequences were *PfGR*_F 5’-GCTGCCTCAGTTCATGATATTT-3’ and *PfGR*_R 5’-CTTATCCCTTCTCTCTACCAACAG-3’. The sequence for the fluorophore labelled probe was /56-FAM/TC CAG GCA T/ZEN/T ACG GAT TTG ACA CCA /3IABkFQ/. *PfGR* was amplified from NF54 genomic DNA and cloned into a pUC19 backbone to generate standard curves of known copy number. Each qPCR reaction contained 3 µL of template DNA, and a reaction master mix containing 5 µL PrimeTime Gene Expression Master Mix without ROX dye (QIAGEN, USA), 0.3 µM of each primer, and 0.1 µM of each probe. The thermal profile used for qPCR was: 1 min at 95 °C; 45 cycles of 15 s at 95 °C and 1 min 60 °C. All qPCR assays were run as technical duplicates and each plate included a no template control, a negative control (uninfected *An. gambiae* DNA) and a positive control (a linearized plasmid containing the *PfGR* amplicon sequence). All assays were run on an Eco™ Real-Time PCR System (Illumina).

### RNAi gene silencing

Double strand RNA (dsRNA) was produced from a fragment of *AgAkh1*, *AgAkh2, AgAkhR* and *GFP* (as a negative control) with the universal T7 polymerase promoter sequence, 5’-TAATACGACTCACTATAGGG-3’, appended to the 5’ of each primer, and cloned into a pJET1.2 plasmid. Primer sequences are given for reference in Supplementary Table S1. The dsRNA was produced using a MEGAscript™ RNAi Kit and purified using a MEGAclear Transcription Clean-Up kit (Ambion). To confirm knockdown efficiency, 5- to 6-day old sugar-fed adult *An. gambiae* females were cold-anesthetized and injected with 69 nL of 5 µg/µL of ds*GFP*, ds*AgAkh1*, ds*AgAkh2*, ds*AgAkhR*, or ds*AgAkh1* + ds*AgAkhR* using a Nanoject II injector (Drummond). Nine females from each treatment were sampled 1-, 3-, and 5-days post dsRNA injection, starved for 3 h (to induce *AgAkh* expression) and preserved in pools of 3 in TRIzol for further analysis. The silencing efficiencies of each dsRNA was determined using RT-qPCR and an *AgAkh1* peptide ELISA. To determine the role of AKH signaling in *P. falciparum* sporogony, *An. gambiae* females were treated with ds*AgAkh1*, ds*AgAkh1* + ds*AgAkhR* and ds*GFP* 3 dpib. A subset of mosquitoes was sampled 24 h post dsRNA injection to confirm knockdown using RT-qPCR and *AgAkh1* ELISA analyses. The rest were sampled at 8 dpib for midgut oocyst enumeration and diameter measurements, and 14 dpib for salivary gland sporozoite enumeration. For oocyst diameter measurements, each midgut was imaged using a Keyence BZ-X700, ensuring oocysts in all focal planes were visible. ImageJ and Progress Capture Pro software were used to measure the diameter of the first 25 oocysts from the anterior end of each midgut. Diameters of all the oocysts in midguts with less than 25 oocysts were measured. Three biological replicates representing three independent infection assays were performed.

### Peptide hormone injections

Synthetic *Ag*Akh1 (pE-LTFTPAW-amide, purity >95%) and *Ag*Akh2 (pE-VTFSRDWNA-amide, purity >95%) were purchased from New England Peptide^TM^. Dilutions of 1.69 pmol/µL (for a final dilution of 116.61 fmol/ mosquito) and 1.16 pmol/µL (for a final dilution of 80.04 fmol/ mosquito) of each peptide were prepared in 1X PBS from stocks of *Ag*Akh1 and *Ag*Akh2, respectively. Each female *An. gambiae* was intrathoracically injected with 69 nL of one of the two peptides using a Nanoject II injector (Drummond). Control groups were injected with 69 nL of PBS. To confirm the effect of the synthetic peptides on downstream genes in the AKH pathway (*AgAkhR, AgLsdp1*, and *AgLp*), 9 females from each treatment and control group were sampled 3 hours postinjection, preserved in pools of 3 in TRIzol and analyzed by RT-qPCR. To determine the effect of the synthetic AKH peptides on *P. falciparum* sporogony, infected *An. gambiae* were intrathoracically injected with synthetic *Ag*Akh1 and *Ag*Akh2 at 3 or 5 dpi. Controls included *P. falciparum-infected* mosquitoes injected with sterile PBS. Mosquitoes were dissected 8 dpib for oocyst enumeration and diameter measurements, and 14 dpib for salivary gland sporozoite quantification. Three biological replicates representing three independent infection assays were performed.

### Harvesting *P. falciparum*-infected midgut supernatant

*Anopheles gambiae* were infected with *P. falciparum* at 1% gametocytemia and 50% hematocrit via SMFA as described above. Mosquitoes were then maintained on 10% sugar solution until day 3, 4, or 5 dpib when midguts were dissected in RPMI media (RPMI 1640 Medium, no glutamine, no phenol red, Gibco^TM^) and pooled as groups of three. Midgut pools were incubated for 24 h in 96-well cell culture plates (CELLTREAT Scientific) with either: (a) 200 µL of media comprising RPMI + 1.25% penicillin-streptomycin + 0.125% amphotericin, or (b) 200 µL of media comprising RPMI + 25% heat-inactivated lipid-rich FBS + 1.25% penicillin-streptomycin + 0.125% amphotericin. Controls included RPMI media only and supernatant from pooled midguts of age/time point-matched non-infectious blood-fed females incubated in both sets of media. Supernatant was carefully pipetted from each cell culture well into 1.5 mL low-bind Eppendorf tubes containing 10 µL protease inhibitor cocktail, snap-frozen in liquid nitrogen, and stored at −80 °C. The midguts from the *P. falciparum*-infected pools were analyzed for parasite burden using the *PfGR* DNA procedure as described above. Only pools that were positive for *P. falciparum* infection were used in the subsequent assays. Another subset was dissected at 8 dpi for oocyst enumeration by microscopy. To increase the supernatant concentration for injection, pools were further combined (*n* = 9 midgut supernatants total) and concentrated to a volume of 200 µL via vacuum centrifuge. Sixty-nine nanoliters of each concentrate was then injected into 5- to 6-day old sugar-fed female *An. gambiae* using a Nanoject II. Mosquitoes were sampled 1 h postinjection, pooled into groups of three and extracted for total RNA as aforementioned. An aliquot of the extracted RNA was subjected to RT-qPCR analysis to detect *AgAkh1, AgAkh2, AgAkhR, AgLsdp1*, and *AgLp* transcripts as per the conditions described above. Another subset of 9 supernatant-injected females were snap frozen in pools of 3 in liquid nitrogen for ELISA analysis. Three biological replicates representing supernatants from 3 independent infectious feeds injected into 3 different cohorts of sugar-fed mosquitoes was conducted.

### Sample preparation for measuring *Ag*Akh1 peptide levels

#### Peptide extraction

Mosquito samples stored from starvation, non-infectious blood feeding, *P. falciparum* blood feeding time points as well as RNAi assays, and midgut supernatant treatments were homogenized in 100 µL acidified methanol (90/9/1 MeOH: CH_3_OOH: H_2_O), each group included three biological replicates with a pool of 3 female mosquitoes. The homogenate was sonicated for 10 min and centrifuged at 16,110 x g at 4 °C for 20 min. The supernatant was transferred to a new 1.5 mL Eppendorf tube. The resultant tissue pellet was rinsed two more times with acidified methanol and combined with the collected supernatant. The combined supernatant was desiccated in a SpeedVac Concentrator and redissolved in 400 µL 1X PBS. Total peptide content was estimated using a Nanodrop 2000 spectrophotometer and used to normalize across the samples.

#### Generation and purification of Anti-AgAkh1 antibodies

*Ag*Akh1 peptide ({pGlu}LTFTPAW) conjugated to KLH via the C-terminus was used to immunize two rabbits to generate polyclonal antibodies. The antibodies were generated by GenScript (Piscataway, NJ, USA), following their standard polyclonal antibody production protocol. Due to low antigenicity of this epitope, a total of five boosts were carried out. Anti-*Ag*Akh1 specific antibodies were purified from the rabbit anti-sera using {pGlu}LTFTPAWCOOH peptide (New England Peptide, Gardner, MA, USA) conjugated to Dynabeads™ M-270 Epoxy (Thermo Fisher Scientific) following manufacturer’s instructions. The IgG titers of the purified Anti-*Ag*Akh1 antibodies was determined using Easy-Titer™ Rabbit IgG assay kit (Thermo Fisher Scientific).

### *Ag*Akh1 competitive peptide Enzyme-Linked Immunosorbent Assay

Maxisorp 96-well ELISA plates (Nunc, Fisher Scientific) were incubated and covered overnight at 4 °C with 100 µL *Ag*Akh1 peptide at 1 μg/mL in 1X PBS. Plates were washed three times with PBS-Tween 20 (0.1%) (PBST20) and blocked with 1% bovine serum albumin (BSA)/1X PBS for 1 h at room temperature. For each ELISA, standard curves were generated using ten-fold dilutions of the *Ag*Akh1 peptide (0, 0.001, 0.01, 0.1, 1, 10, 100 µg/mL). The standards and the mosquito samples (described above) were spiked with Anti-*Ag*Akh1 Abs at 5 µg/mL and incubated for 10 min at room temperature, and 100 µL of each standard/sample was then added into wells in triplicates, covered, and incubated at room temperature for 1 h. Plates were then washed three times with PBST20 and incubated with 100 μL of a horseradish peroxidase (HRP)-conjugated anti-Rabbit IgG (Thermo Scientific) diluted to 1:5,000 in 0.5% BSA/ 1X PBS, covered and incubated for 1 h at room temperature. Plates were then washed again three times with PBST20 and 100 μL of TMB (3,3′,5,5′-tetramethylbenzidine) microwell peroxidase substrate (Kirkegaard & Perry Laboratories) was added to each well. The reaction was stopped after five min by adding 100 μL of 1 M phosphoric acid. The optical density was read at 450 nm and 570 nm using SynergyMx Multi-Mode Microplate Reader. Background was corrected for by subtracting the OD value at 450 nm from the OD value at 570 nm. A standard curve was generated and used to estimate the amount of *Ag*Akh1 peptide under each different treatment condition.

### Statistics and reproducibility

The statistical methods used to analyze each experiment are detailed in the figure legends. The cycles to threshold (Ct) values of each sample were used to generate relative gene expression levels and normalized to *AgRPL32* which does not change with starvation, blood feeding and infection. Fold change in expression levels between different treatments was determined by double delta Ct (−∆∆Ct). Resultant transcriptomic expression data was subjected to a Shapiro test for normality and Levine’s test of homogeneity, and transformations to generate normality before non-parametric tests were attempted. The lme4 (v 1.1)^52^, and multcomp (v 1.4)^53^ packages were used to run linear mixed-effects models analyzing the differences between sporozoite genome copy number and oocyst diameter data, using treatment (PBS, S*AgAkh1*, S*AgAkh2*, ds*GFP*, ds*AgAkh1*) as a fixed effect and biological replicate as a random effect. These packages were also used to analyze oocyst intensities between treatments and their respective controls using a zero-inflated generalized linear model at a 95% confidence interval. Differences in peptide titers were detected by a Kruskal-Wallis test and two-sample Wilcoxon test. Midgut oocyst and salivary gland sporozoite data were analyzed using Chi-square test. Effect size of dsRNA and synthetic peptide treatments on oocyst diameter and sporozoite genome copy number was estimated using Cohen’s *d* effect size. Similarly, the effect size of midgut supernatant treatment on peptide levels were estimated using Cohen’s *d* effect size. Statistical analyses were completed in GraphPad Prism 7 and R version 3.6.3^54^. All statistical tests were analyzed at an α = 0.05.

### Reporting summary

Further information on research design is available in the Nature Portfolio Reporting Summary linked to this article.

## Supplementary information

Supplementary Information

Description of Additional Supplementary Files

Supplementary Data 1

Reporting Summary

## Data Availability

The source data for all graphs and charts are provided in Supplementary Data 1 and Figshare (10.6084/m9.figshare.21771071.v1).
