# Supplementary material for: Adipokinetic hormone signaling in the malaria vector Anopheles gambiae facilitates Plasmodium falciparum sporogony
Source: Commun Biol. 2023 Feb 13;6:171. doi: 10.1038/s42003-023-04518-6 (PMC9924834; doi:10.1038/s42003-023-04518-6)
Supplement: Supplementary file 1 — Supplementary Information [file 42003_2023_4518_MOESM1_ESM.pdf]

Supplementary Information for
Adipokinetic hormone signaling in the malaria vector *Anopheles gambiae* promotes *Plasmodium falciparum* sporogony.

Vincent O. Nyasembe[1], Timothy Hamerly[1], Borja López-Gutiérrez[1], Alexandra M. Leyte-Vidal[1], Heather Coatsworth[1], Rhoel R. Dinglasan[1,*]

[1]Department of Infectious Diseases and Immunology, College of Veterinary Medicine & Emerging Pathogens Institute, University of Florida, 2055 Mowry Road, Gainesville, FL 32611, United States of America

Rhoel R. Dinglasan
Email: rdinglasan@epi.ufl.edu

This PDF file includes:

Figure S1
Tables S1 and S2

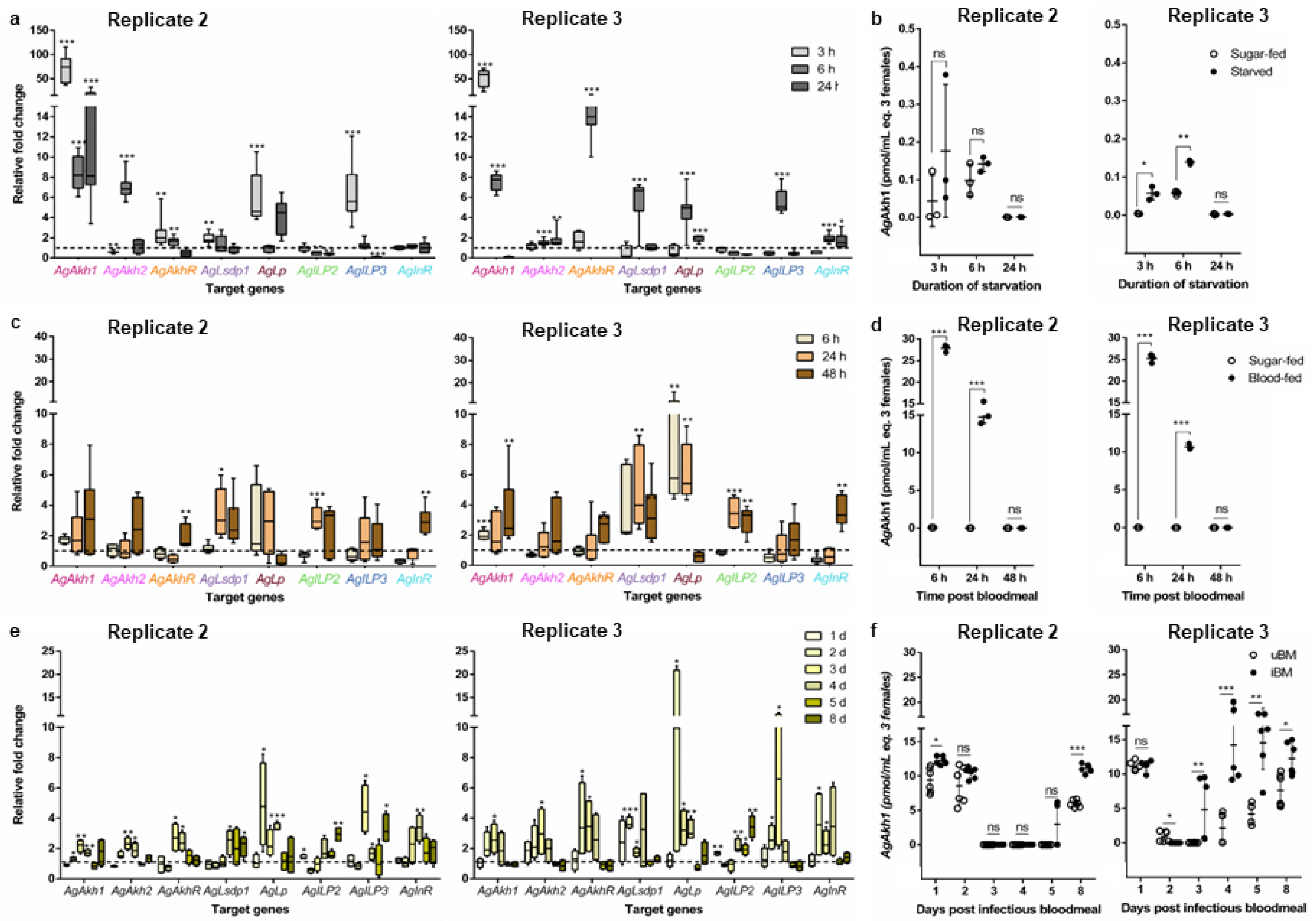

Supplementary Fig. S1. Expression profiles of gene transcripts and peptide in AKH signaling and Insulin/insulin-like Growth Factor signaling (IIS) pathways in starved and blood-fed *An. gambiae.* (a) Replicates 2 and 3 showing time-dependent fold change in expression of AKH (*AgAkh1, AgAkh2, AgAkhR, AgLsdp1,* and *AgLp*) and IIS (*AgILP2, AgILP3 and AgInR*) pathway genes during starvation relative to age-matched sugar-fed females. All expression levels were normalized against the control gene 60S ribosomal protein L32 (*AgRpL32*). Sugar-fed expression levels were standardized to 1 (dotted line). Bars not capped with asterisk were not statistically different from sugar-fed controls. (**b**) Representative replicates 2 and 3 showing *Ag*Akh1 peptide titers in sugar-fed and starved female *An. gambiae* at different time points detected by competitive peptide ELISA. (**c**) Replicates 2 and 3 showing time-dependent fold change in expression of AKH and IIS pathways genes following blood feeding relative to age-matched sugar-fed females. All expression levels were normalized against the control gene 60S ribosomal protein L32 (*AgRpL32*). Sugar-fed expression levels were standardized to 1 (dotted line). Bars not capped with asterisk were not statistically different from sugar-fed controls. **(d)** Replicates 2 and 3 showing *Ag*Akh1 peptide titers in sugar-fed and blood-fed female *An. gambiae* at different time points post bloodmeal. N = 3 pools of 3 mosquitoes for each treatment at each time point. (**e**) Replicates 2 and 3 showing time-dependent fold change in expression of AKH and IIS pathways genes in *P. falciparum*-infected *An. gambiae* relative to uninfected mosquitoes at different time points. All expression levels were normalized against the control gene 60S ribosomal protein L32 (*AgRpL32*). Uninfected blood-fed expression levels were standardized to 1 (dotted line). Bars not capped with asterisk were not statistically different from sugar-fed controls. (**f**) Replicates 2 and 3 showing *Ag*Akh1 peptide titers in sugar-fed and blood-fed female *An. gambiae* at different time points post infectious bloodmeal. *P. falciparum* oocyst prevalence and intensities in replicates 2 and 3 were 95% and 55.5; and 90% and 67, respectively. Error bars represent standard error of mean. Differences in gene expression between infected and uninfected mosquitoes were detected by independent samples t-test or two-sample Wilcoxon test at 95% confidence interval. * < 0.05, ** > 0.01, *** > 0.001, ns = not significant (*P* > 0.05). A total of 9 mosquitoes pooled in groups of three were analyzed for each time point per for each feeding status.

Supplementary Table S1. Oligonucleotide primer sequences for qPCR and dsRNA synthesis.

Name	Gene	Oligonucleotide sequence	Size (bp)
AgAkh1	AGAP008834	5'-CAAGGTGCGATGGGAATTAATC-3'	130
		5'-GGCTGCAATCAACAATCTTCTG-3'	
AgAkh2	AGAP002430	5'-TGAATGCTCTGCCATCTGG-3'	146
		5'-TGTTCCCGCTGTTGCTC-3'	
AgAkhR	AGAP002156	5'-ACGCTACGGATGACCATAATG-3'	131
		5'-AGTCCCTTCTGTATTCGTTGG-3'	
AgLsdp1	AGAP002890	5'-GAAATCGTGCTGTGCATTACC-3'	146
		5'-TCTTGTACGGGCTTTCATCTG-3'	
AgLp	AGAP001826	5'-GGATAAGGAAGAGTTCGCTACG-3'	117
		5'-GCCGACTTTAGTTCACCATTTC-3'	
AgILP1	AGAP010603	5'-GCAACAGATTCAATGGATTCCG-3'	151
		5'-GCCTGGTAGATCATGTCCG-3'	
AgILP2	AGAP010600	5'-GATCAGCCAGCTAACACGCT-3'	143
		5'- TGGTTTGCCTGATCGTGTAC-3'	
AgILP3	AGAP010602	5'-GGTAAAGGTACTGTCCTTCCTG-3'	141
		5'-AGTATCTGCTGCGTGTTGTC-3'	
AgILP4	AGAP010601	5'-TGCCGAAGATACTTGCCATG-3'	140
		5'-CCCATTCTCACTAAAGTCCCAG-3'	
AgILP5	AGAP003927	5'-GTTTGCCGACATTGTCAGTG-3'	139
		5'-ATGGCGTATCAGTTTCTCCC-3'	
AgILP6	AGAP010604	5'-GGTCCAGGTTTGCAGTACAG-3'	143
		5'-AACGCTTGCAGGATGTAGAG-3'	
AgILP7	AGAP010605	5'-GCAACAGATTCAATGGATTCCG-3'	151
		5'-GCCTGGTAGATCATGTCCG-3'	
AgInR	AGAP012424	5'-TGCTTTACAGACTACAACCCG-3'	140
		5'-CCACCCGACAGTTTCTTAGTC-3'	
AgRPL32	AGAP002122	5'-ATCGCTATGATAAACTCGCCC-3'	147
		5'-ATGTTCGGCATCAGGTACTG-3'	
ds*AgAkh1*	AGAP008834	5'-taatacgactcactatagggTGGATACCGTGAAGCTGTTC-3'	236
		5'-taatacgactcactatagggCTTCTGGCTGCAATCAACAA-3'	
ds*AgAkhR*	AGAP	5'-taatacgactcactatagggTCGTCGCGGATCAATATCAT-3'	222
		5'-taatacgactcactatagggCGGTTTCAGCACAGCAAAGT-3'	

Akh = Adipokinetic hormone, *AKHR* = adipokinetic hormone receptor, *Lsdp1* = Lipid storage droplets surface-binding protein 1, *Lp* = lipophorin, *ILP* = insulin-like peptide, *InR* = insulin receptor, *RPL*32 = ribosomal protein L32. Lowercase oligonucleotide sequence = T7 promoter.

Supplementary Table S2. Relative expression of Insulin/insulin-like growth signaling pathway genes during starvation, following blood feeding, and during *Plasmodium falciparum* sporogony.

	**Starvation**		
	**Fold change in gene expression relative to sugar-fed females ± SE (*P*-value)**		
**Gene**	**3 h**	**6 h**	**24 h**
AgILP1	1.47 ± 0.34 (0.238)	0.42 ± 0.19 (0.151)	0.86 ± 0.32 (0.68)
AgILP4	1.26 ± 0.29 (0.414)	1.7 ± 0.55 (0.715)	1.20 ± 0.23 (0.436)
AgILP5	0.99 ± 0.09 (0.889)	3.31 ± 1.32 (0.249)	0.89 ± 0.25 (0.718)
AgILP6	1.22 ± 0.10 (0.091)	1.62 ± 0.19 (0.067)	0.98 ± 0.20 (0.938)
AgILP7	1.15 ± 0.18 (0.473)	1.64 ± 0.15 (0.051)	0.93 ± 0.17 (0.722)

	**Blood feeding**		
	**Fold change in gene expression relative to sugar-fed females ± SE (*P*-value)**		
**Gene**	**6 h**	**24 h**	**48 h**
AgILP1	0.78 ± 0.14 (0.197)	0.73 ± 0.12 (0.126)	0.86 ± 0.13 (0.407)
AgILP4	1.12 ± 0.19 (0.562)	1.36 ± 0.08 (0.006)	1.23 ± 0.14 (0.155)
AgILP5	0.54 ± 0.16 (<0.061)	0.79 ± 0.23 (0.439)	2.20 ± 0.48 (0.052)
AgILP6	0.74 ± 0.12 (0.115)	1.80 ± 0.59 (0.230)	1.47 ± 0.21 (0.074)
AgILP7	0.88 ± 0.32 (0.744)	1.43 ± 0.40 (0.335)	1.44 ± 0.59 (0.496)

	***P. falciparum* infection**					
	**Fold change in gene expression relative to uninfected females ± SE (*P*- value)**					
**Gene**	**1 d**	**2 d**	**3 d**	**4 d**	**5 d**	**8 d**
AgILP1	0.97 ± 0.04 (0.592)	1.19 ± 0.12 (0.376)	3.34 ± 0.49 (0.015)	1.14 ± 0.16 (0.464)	0.99 ± 0.17 (0.972)	2.81 ± 0.69 (0.078)
AgILP4	1.11 ± 0.11 (0.424)	1.01 ± 0.12 (0.950)	2.23 ± 0.72 (0.195)	3.06 ± 1.76 (0.330)	1.24 ± 0.13 (0.164)	3.24 ± 1.00 (0.111)
AgILP5	1.05 ± 0.24 (0.839)	0.82 ± 0.07 (0.108)	2.78 ± 0.55 (0.044)	1.21 ± 0.24 (0.457)	0.99 ± 0.09 (0.987)	3.40 ± 0.74 (0.0472)
AgILP6	1.08 ± 0.15 (0.731)	2.27 ± 0.55 (0.118)	3.08 ± 0.29 (0.004)	1.96 ± 0.24 (0.023)	1.30 ± 0.16 (0.164)	3.18 ± 0.90 (0.095)
AgILP7	0.97 ± 0.09 (0.857)	2.13 ± 0.64 (0.177)	3.44 ± 0.72 (0.041)	1.35 ± 0.16 (0.147)	0.98 ± 0.05 (0.881)	2.51 ± 0.68 (0.112)

Gene expressions were normalized to 60S ribosomal protein L32 (*AgRpL32*). Differences in gene expression between sugar-fed and starved, sugar-fed and blood-fed mosquitoes, and *P. falciparum* infected and uninfected mosquitoes were detected by independent samples t-test or two-sample Wilcoxon test at 95% confidence interval. The time points represent hours of starvation (h), hours post bloodmeal (h), and days post infectious bloodmeal (d). ILP = insulin-like peptide. Three biological replicates were conducted.
