# Supplementary material for: Adipokinetic hormone signaling in the malaria vector Anopheles gambiae facilitates Plasmodium falciparum sporogony
Source: Commun Biol. 2023 Feb 13;6:171. doi: 10.1038/s42003-023-04518-6 (PMC9924834; doi:10.1038/s42003-023-04518-6)
Supplement: Supplementary file 4 — Reporting Summary [file 42003_2023_4518_MOESM4_ESM.pdf]

nature portfolio

Corresponding author(s): Rhoel R. Dinglasan

Last updated by author(s): Apr 5, 2022

Reporting Summary

Nature Portfolio wishes to improve the reproducibility of the work that we publish. This form provides structure for consistency and transparency in reporting. For further information on Nature Portfolio policies, see our Editorial Policies and the Editorial Policy Checklist.

Statistics

For all statistical analyses, confirm that the following items are present in the figure legend, table legend, main text, or Methods section.

n/a	Confirmed	
☐	☒	The exact sample size (*n*) for each experimental group/condition, given as a discrete number and unit of measurement
☐	☒	A statement on whether measurements were taken from distinct samples or whether the same sample was measured repeatedly
☐	☒	The statistical test(s) used AND whether they are one- or two-sided<br>*Only common tests should be described solely by name; describe more complex techniques in the Methods section.*
☐	☒	A description of all covariates tested
☐	☒	A description of any assumptions or corrections, such as tests of normality and adjustment for multiple comparisons
☐	☒	A full description of the statistical parameters including central tendency (e.g. means) or other basic estimates (e.g. regression coefficient) AND variation (e.g. standard deviation) or associated estimates of uncertainty (e.g. confidence intervals)
☐	☒	For null hypothesis testing, the test statistic (e.g. *F*, *t*, *r*) with confidence intervals, effect sizes, degrees of freedom and *P* value noted<br>*Give P values as exact values whenever suitable.*
☒	☐	For Bayesian analysis, information on the choice of priors and Markov chain Monte Carlo settings
☒	☐	For hierarchical and complex designs, identification of the appropriate level for tests and full reporting of outcomes
☐	☒	Estimates of effect sizes (e.g. Cohen's *d*, Pearson's *r*), indicating how they were calculated

Our web collection on statistics for biologists contains articles on many of the points above.

Software and code

Policy information about availability of computer code

Data collection	qPCR data was obtained using Eco™ Real-Time software, and ELISA data was collected using SynergyMx Gen5 software.
Data analysis	Statistical analyses were completed in GraphPad Prism 7 and R version 3.6.3.

For manuscripts utilizing custom algorithms or software that are central to the research but not yet described in published literature, software must be made available to editors and reviewers. We strongly encourage code deposition in a community repository (e.g. GitHub). See the Nature Portfolio guidelines for submitting code & software for further information.

Data

Policy information about availability of data

All manuscripts must include a data availability statement. This statement should provide the following information, where applicable:

- Accession codes, unique identifiers, or web links for publicly available datasets
- A description of any restrictions on data availability
- For clinical datasets or third party data, please ensure that the statement adheres to our policy

All relevant raw data is included in the manuscript as a supplementary data file.

Field-specific reporting

Please select the one below that is the best fit for your research. If you are not sure, read the appropriate sections before making your selection.

☒ Life sciences ☐ Behavioural & social sciences ☐ Ecological, evolutionary & environmental sciences

For a reference copy of the document with all sections, see nature.com/documents/nr-reporting-summary-flat.pdf

Life sciences study design

All studies must disclose on these points even when the disclosure is negative.

Sample size	Pooled sample sizes were determined using the minimum amount of tissue needed for downstream sample processing. Replicate numbers were considered sufficient based on previously published studies in the discipline.
Data exclusions	No data was excluded.
Replication	All experiments were replicated three times.
Randomization	Allocation of organisms into experimental groups was pseudo-random – female mosquitoes were sorted from males in no particular order and allocated into cups, which were hen assigned to treatments – also in no particular order.
Blinding	Blinding was not performed due to personnel limitations.

Reporting for specific materials, systems and methods

We require information from authors about some types of materials, experimental systems and methods used in many studies. Here, indicate whether each material, system or method listed is relevant to your study. If you are not sure if a list item applies to your research, read the appropriate section before selecting a response.

Materials & experimental systems

n/a	Involved in the study	
☐	☒	Antibodies
☒	☐	Eukaryotic cell lines
☒	☐	Palaeontology and archaeology
☐	☒	Animals and other organisms
☒	☐	Human research participants
☒	☐	Clinical data
☒	☐	Dual use research of concern

Methods

n/a	Involved in the study	
☒	☐	ChIP-seq
☒	☐	Flow cytometry
☒	☐	MRI-based neuroimaging

Antibodies

Antibodies used	Anti-AgAkh1 specific antibodies (GenScript).
Validation	The IgG titers of the purified Anti-AgAkh1 antibodies was determined using Easy-Titer™ Rabbit IgG assay kit (Thermo Fisher Scientific).

Animals and other organisms

Policy information about studies involving animals; ARRIVE guidelines recommended for reporting animal research

Laboratory animals	Adult female Anopheles gambiae (Keele) mosquitoes.
Wild animals	N/A
Field-collected samples	N/A
Ethics oversight	No ethics approval is required for invertebrates.

Note that full information on the approval of the study protocol must also be provided in the manuscript.
